# Immune system adaptation during gender-affirming testosterone treatment

**DOI:** 10.1038/s41586-024-07789-z

**Published:** 2024-09-04

**Authors:** Tadepally Lakshmikanth, Camila Consiglio, Fabian Sardh, Rikard Forlin, Jun Wang, Ziyang Tan, Hugo Barcenilla, Lucie Rodriguez, Jamie Sugrue, Peri Noori, Margarita Ivanchenko, Laura Piñero Páez, Laura Gonzalez, Constantin Habimana Mugabo, Anette Johnsson, Henrik Ryberg, Åsa Hallgren, Christian Pou, Yang Chen, Jaromír Mikeš, Anna James, Per Dahlqvist, Jeanette Wahlberg, Anders Hagelin, Mats Holmberg, Marie Degerblad, Magnus Isaksson, Darragh Duffy, Olle Kämpe, Nils Landegren, Petter Brodin

**Affiliations:** 1 Department of Women’s and Children’s Health, Karolinska Institutet, Solna, Sweden; 2 Department of Laboratory Medicine, Lund University, Lund, Sweden; 3 Center for Molecular Medicine, Department of Medicine, Karolinska Institutet, Solna, Sweden; 4 Science for Life Laboratory, Department of Medical Biochemistry and Microbiology, Uppsala University, Uppsala, Sweden; 5 Translational Immunology Unit, Institut Pasteur, Paris, France; 6 Department of Clinical Chemistry, Sahlgrenska University Hospital, Gothenburg, Sweden; 7 Department of Internal Medicine and Clinical Nutrition, University of Gothenburg, Gothenburg, Sweden; 8 Department of Public Health and Clinical Medicine, Umeå University, Umeå, Sweden; 9 Department of Medicine, Örebro University, Örebro, Sweden; 10 ANOVA, Karolinska University Hospital, Stockholm, Sweden; 11 Department of Medicine, Karolinska Institutet, Stockholm, Sweden; 12 Department of Molecular Medicine and Surgery, Karolinska Institutet, Solna, Sweden; 13 Department of Medical Sciences, Uppsala University, Uppsala, Sweden; 14 Department of Endocrinology, Metabolism and Diabetes, Karolinska University Hospital, Stockholm, Sweden; 15 Medical Research Council, Laboratory of Medical Sciences, London, UK; 16 Department of Immunology and Inflammation, Imperial College London, London, UK

**Keywords:** Translational research, Epigenetics in immune cells

## Abstract

Infectious, inflammatory and autoimmune conditions present differently in males and females. SARS-CoV-2 infection in naive males is associated with increased risk of death, whereas females are at increased risk of long COVID^1^, similar to observations in other infections^2^. Females respond more strongly to vaccines, and adverse reactions are more frequent^3^, like most autoimmune diseases^4^. Immunological sex differences stem from genetic, hormonal and behavioural factors^5^ but their relative importance is only partially understood^6–8^. In individuals assigned female sex at birth and undergoing gender-affirming testosterone therapy (trans men), hormone concentrations change markedly but the immunological consequences are poorly understood. Here we performed longitudinal systems-level analyses in 23 trans men and found that testosterone modulates a cross-regulated axis between type-I interferon and tumour necrosis factor. This is mediated by functional attenuation of type-I interferon responses in both plasmacytoid dendritic cells and monocytes. Conversely, testosterone potentiates monocyte responses leading to increased tumour necrosis factor, interleukin-6 and interleukin-15 production and downstream activation of nuclear factor kappa B-regulated genes and potentiation of interferon-γ responses, primarily in natural killer cells. These findings in trans men are corroborated by sex-divergent responses in public datasets and illustrate the dynamic regulation of human immunity by sex hormones, with implications for the health of individuals undergoing hormone therapy and our understanding of sex-divergent immune responses in cisgender individuals.

## Abstract (web summary)

Examination of immunological changes in transgender individuals undergoing gender-affirming testosterone treatment reveals sex hormone-regulated pathways in humans and explains sex-divergent responses in cisgender individuals.

## Main

**Fig. 1:**
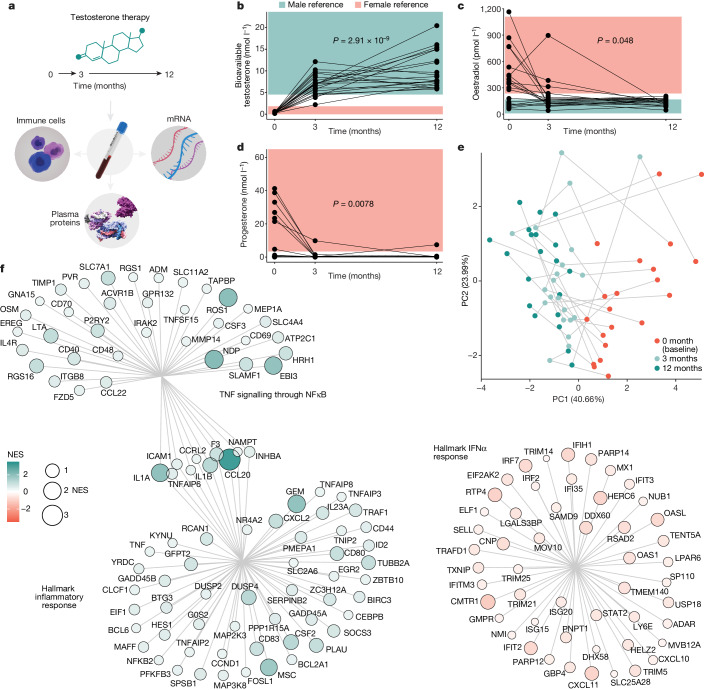
Immunological investigation in individuals undergoing gender-affirming testosterone therapy. **a**, Systems-level assessment of blood immune system in individuals assigned female sex at birth (trans men) in blood samples collected at baseline, and after 3 and 12 months of oral testosterone therapy (*n* = 23). **b**–**d**, Sex hormone concentrations measured in serum samples (*n* = 66) using liquid chromatography with tandem mass spectrometry in a single experiment and shown in relation to female (pink) and male (blue) reference ranges before and during testosterone therapy. Kruskal–Wallis tests (5% false discovery rate (FDR) corrected) for bioavailable testosterone (**b**), oestradiol (**c**) and progesterone (**d**). **e**, PCA on the basis of nine sex hormones, first two principal components (PC1 and PC2; percentage variance explained) and sample points coloured by sample timepoint. **f**, Bulk RNA-seq from whole blood samples (*n* = 60) and differently expressed mRNA transcripts analysed by normalized enrichment scores (NES) for Hallmark pathways. Hallmark IFNα responses decrease after 12 months of testosterone treatment, TNF signalling through NFκB and Hallmark inflammatory responses increased after 12 months of testosterone treatment as compared with baseline.

**Extended Data Fig. 1:**
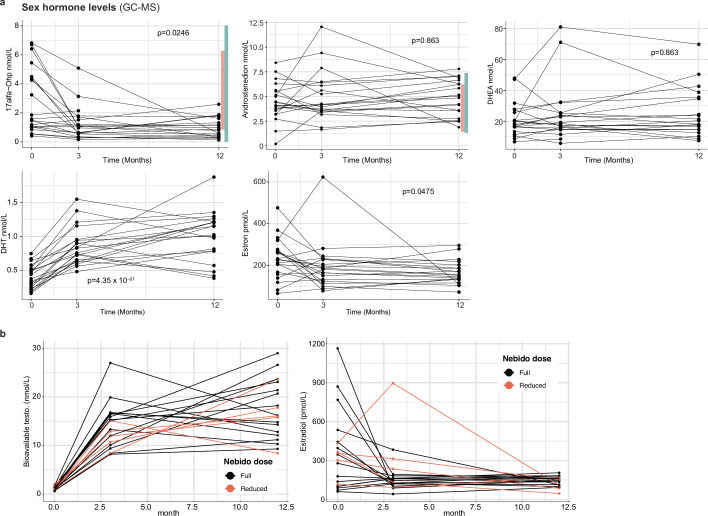
Modeling of whole blood mRNA transcriptome data. Sex hormone measurements by LCMS over time during testosterone treatment in n = 22 subjects and 3 timepoints per subject. Shaded areas represent male (blue) and female (pink) reference ranges where available. P-values from Kruskal-Wallis tests (5% FDR corrected) **b**) Testosterone and estradiol levels in patients receiving full dose (1000 mg, black) or reduced doses (750 mg, orange) of Nebido at one or more timepoints.

Gender-affirming hormone therapy (GAHT) enables the acquisition of secondary sex characteristics better aligned with gender identity in transgender individuals. It is important to understand how GAHT influences the immune response in these individuals, but this also provides a unique opportunity to investigate the immunomodulatory functions of gonadal steroids in vivo in humans of reproductive age. We performed longitudinal blood sampling of 23 trans men, who were assigned female sex at birth and undergoing masculinizing treatment with testosterone undecaonate starting at the age of 18–37 years. Blood samples were collected at baseline and following 3 and 12 months of testosterone treatment (Fig. 1a). By analysing plasma proteins, immune cell phenotypes and functional immune cell responses in vitro, we searched for coordinated changes among cell populations and the protein mediators by which these communicate to understand global changes in response to testosterone treatment. Serum concentrations of bioavailable testosterone increased to male reference range values (Fig. 1b), whereas oestradiol concentrations decreased from baseline to 3 months (Fig. 1c), as did progesterone concentrations (Fig. 1d). When integrating eight different hormones (Fig. 1b–d and Extended Data Fig. 1a), a shared directionality was observed during testosterone therapy (Fig. 1e). Five individuals received lower doses (Nebido, 750 mg) due to low body mass indices but their plasma hormone concentrations were comparable (Extended Data Fig. 1b). Bulk mRNA sequencing (mRNA-seq) of longitudinal blood samples (*n* = 60 from 20 out of 23 participants) showing decreasing transcripts enriched for Hallmark interferon (IFN)-α (IFNα) responses (Fig. 1f), whereas upregulated transcripts were enriched for the Hallmark pathway of tumour necrosis factor (TNF) signalling through nuclear factor kappa B (NFκB) and Hallmark inflammatory responses (Fig. 1f), indicating a previously unappreciated role for gonadal steroids in calibrating type-I interferon (IFN-I)/TNF cross-regulation.

## Testosterone-induced immune cell changes

**Fig. 2:**
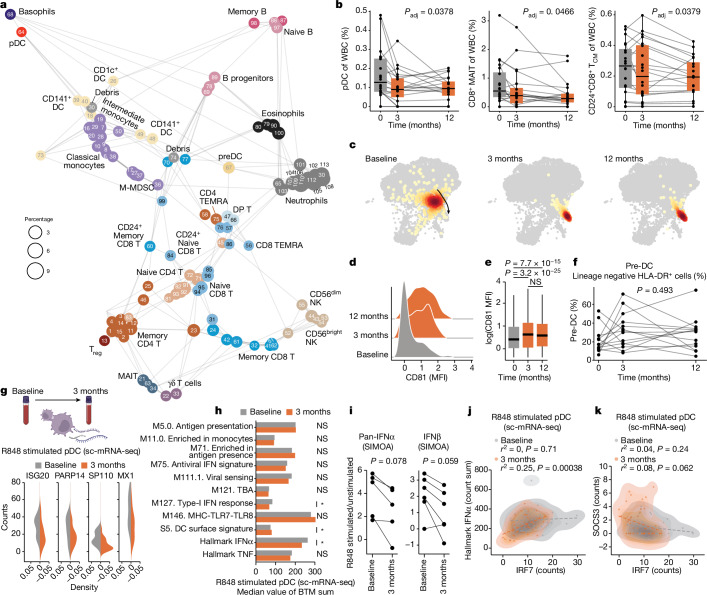
Immune cell changes during gender-affirming testosterone therapy. **a**, Immune cell clusters (FlowSOM) of 12,377,068 cells by 50-marker mass cytometry. *N* = 113 clusters annotated to lineages (*n* = 35). Cluster-IDs match expression heatmap (Extended Data Fig. 2b). **b**, Cell frequencies in *n* = 60 samples from 20 participants, four experiments, linear mixed-effects models with visit and age as fixed and participant as random effect. Boxplot centre, median; maximum, Q3 + 1.5 × IQR (IQR values ranging from Q1 to Q3); minimum whisker, Q1 − 1.5 × IQR; *P* values 5% FDR corrected. **c**, Two-dimensional embedding (ForceAtlas2) of pDCs (*n* = 742) analysed by mass cytometry in a single donor and one representative experiment of four. **d**, CD81 expression in pDCs from samples in **c**. **e**, Summary of CD81 concentrations in pDCs from 18 donors across four experiments (*n* = 15,197). Boxplot centre, median; maximum, Q3 + 1.5 × IQR (IQR values ranging from Q1 to Q3); minimum whisker, Q1 – 1.5 × IQR. Two-sided, uncorrected Wilcoxon rank sum test. **f**, Manually gated pre-DCs in lineage negative, HLA-DR^+^ cells (Extended Data Fig. 2c) in 42 samples, 14 participants from three experiments by one-way analysis of variance. **g**, Normalized counts of four IFN-I response genes in R848-stimulated pDCs by sc-mRNA-seq at baseline (*n* = 41) and 3 months (*n* = 47), of two experiments. Counts in stimulated cells, subtracting median counts in unstimulated pDCs. **h**, Median sums of genes assigned to indicated BTMs in R848-stimulated pDCs by sc-mRNA-seq at baseline and 3 months. *P < 0.05. Uncorrected, two-sided Student’s *t*-test. **i**, pan-IFNα and IFNb protein ratios (R848-stimulated versus unstimulated) in PBMC cultures; *P* values comparing ratios at baseline and 3 months by one-sided, paired Student’s *t*-tests. **j**,**k**, pDC sc-mRNA-seq of R848-stimulated pDCs at baseline (*n* = 41) and 3 months (*n* = 47) in two independent experiments by uncorrected, two-sided Student’s *t*-tests and *R*-values from Pearson correlation coefficients, IRF7 counts versus Hallmark IFNα count sum (**j**), and IRF7 versus SOCS3 counts (**k**). MAIT, mucosa-associated invariant T cells; MFI, mean fluorescence intensity; NS, non-significant.

**Extended Data Fig. 2:**
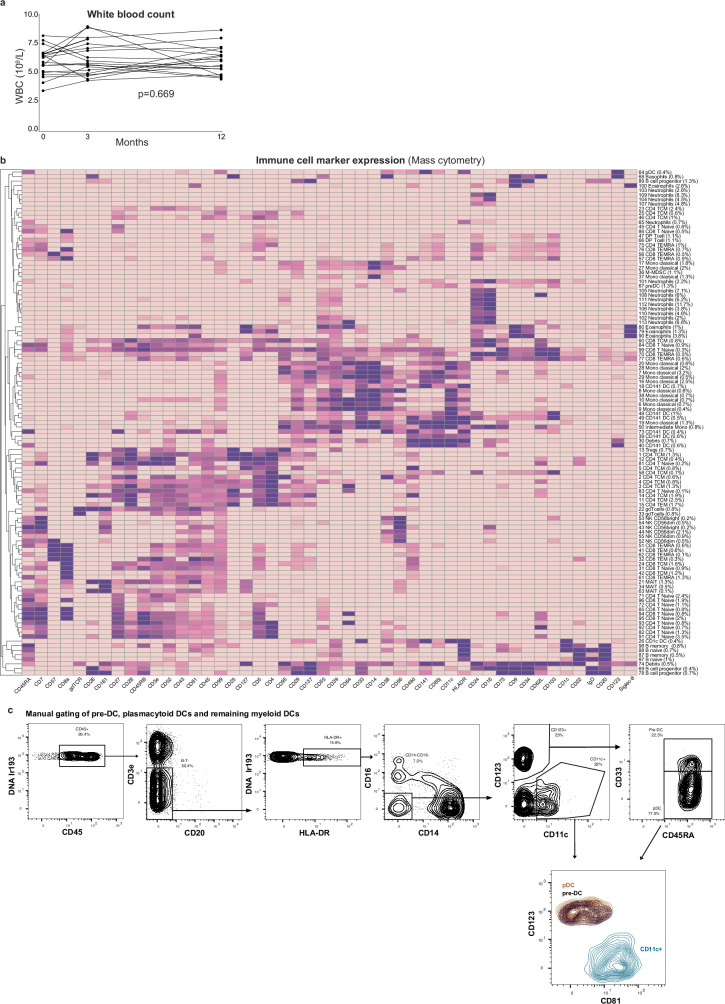
Immune cell changes during gender affirming testosterone therapy. **a**) White blood counts as measured by clinical chemistry analyses at baseline and following 3- and 12-months of testosterone therapy. Repeated measures ANOVA, unadjusted p-value. **b**) Marker expression (Z-score transformed per marker) across all 113 immune cell clusters and n = 12,377,068 cells **c**) Manual gating strategy to identify pDC, pre-DC and CD11c^+^ DC populations among lineage negative HLA-DR^+^ cells analyzed by Mass cytometry.

We stabilized whole blood cells directly at blood collection, stained with a 50-parameter antibody panel and acquired 12,377,068 cells by mass cytometry. There was no significant change in total white blood cell (WBC) counts during testosterone treatment (Extended Data Fig. 2a) and a total of 113 immune cell clusters were identified and embedded in a force-directed graph (Fig. 2a) and annotated manually by median marker expression (Extended Data Fig. 2b). Using mixed-effects models with age and study visit as fixed effects and participant as a random effect, we identified changes in several immune cell populations when comparing samples before and during testosterone treatment. We found an overall contraction of plasmacytoid dendritic cells (DCs) (pDCs), CD8^+^ mucosa-associated invariant T cells and CD24^+^CD8^+^ central memory T cells (T_CM_) during GAHT (Fig. 2b).

## Testosterone-mediated adaptation of pDCs

pDCs are efficient producers of IFN-I, and their contraction can contribute to the reduction in Hallmark IFNα transcripts by testosterone (Fig. 1f). We also found pDC phenotypic changes upon testosterone treatment (Fig. 2c), with surface expression of CD81 on pDCs increasing from 3 months (Fig. 2d,e). CD5^+^CD81^+^ pDCs were reported previously to differ from CD5^−^CD81^−^ pDCs with attenuated type-I IFN responses and more potent regulatory T (T_reg_) cell induction^9^. CD5^+^ DCs have since been shown to differ from classical pDCs and are termed AXL^+^SIGLEC6^+^ DCs (AS-DC)^10^, transitional DCs (tDCs)^11^ or pre-DCs^12^. To study pre-DCs during GAHT, we gated these manually (Extended Data Fig. 2b)^12^ and found no change in abundance during testosterone therapy in vivo (Fig. 2f).

**Extended Data Fig. 3:**
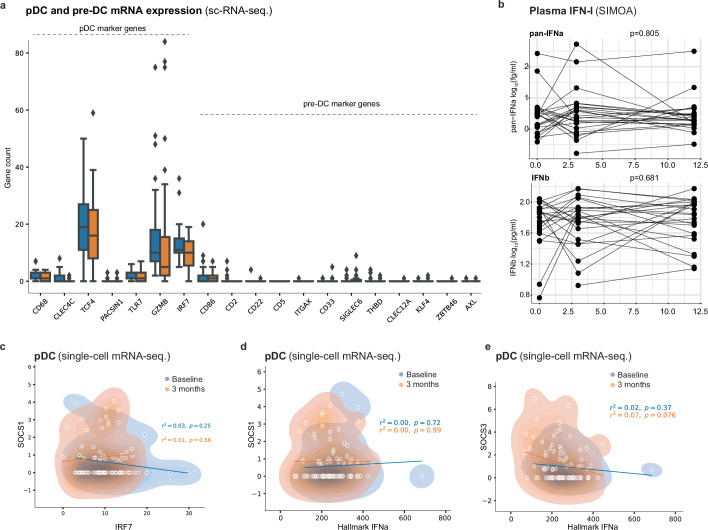
pDC functional responses. **a**) pDc and pre-DC marker genes as reported by Villani et al shown in n = 41 pDCs from baseline and n = 47 pDCs at 3-months from two independent experiments. Boxplot centre = median, max whisker = Q3 + 1.5*IQR (IQR = values ranging from Q1-Q3), min whisker = Q1 – 1.5*IQR. **b**) Plasma pan-IFNα and IFNβ protein levels measured by SIMOA in 71 samples from 24 subjects across two experiments at baseline and following 3 and 12 months of testosterone therapy. Repeated measures ANOVA, unadjusted p-values. **c**) pDC RNA counts for IRF7 vs SOCS1 following R848 stimulation in vitro comparing pDCs collected at baseline (n = 41) and following 3 months of testosterone (n = 47) across two independent experiments. Uncorrected, 2-sided t-tests and R-values from pearson correlation-coefficients, **d**) pDC RNA counts for SOCS1 v.s. Hallmark IFNα following R848 stimulation and, **e**) pDC RNA counts for SOCS3 v.s. Hallmark IFNα following R848 stimulation.

Webb et al. previously reported lower frequencies of IFNα-producing pDCs upon TLR7/8 stimulation in transgender birth females as compared with postpubertal cisgender females^13^. To directly compare pDC functional responses before and after testosterone treatment in transgender birth female participants, we stimulated peripheral blood mononuclear cells (PBMCs) from baseline and after 3 months of testosterone by R848 (TLR7/8) and analysed individual pDCs by single-cell RNA sequencing (scRNA-seq). We verified pDC classification without pre-DC inclusion (Extended Data Fig. 3a)^10^ and found interferon-stimulated genes (ISGs), ISG20, PAPR14, SP110 and MX1 (counts) to be less induced after 3 months of testosterone as compared with baseline (Fig. 2g). This corroborates a recent report of pDC responses in six trans men^14^. When investigating blood transcriptional modules (BTM) and hallmark gene pathways in these R848-stimulated pDC before and during testosterone, BTM *S5* (DC surface signature) was induced, while the Hallmark IFNα gene set and the related M127 IFN-I response, were attenuated significantly (Fig. 2h). We also investigated IFN-I protein secretion and found plasma pan-IFNα concentrations stable (Extended Data Fig. 3b), while pan-IFNα and IFNb concentrations secreted upon R848 stimulation trended lower in PBMC cultures stimulated ex vivo with samples collected after 3 months of testosterone therapy as compared with baseline (Fig. 2i). We conclude that pDCs contract in vivo and adapt phenotypically and functionally, leading to attenuated IFN-I responses during testosterone therapy.

## Regulators of IFN-I responses in pDCs

IRF7 is a master regulator of IFN-I responses in pDCs^15^ and individuals with loss of function mutations in IRF7 fail to control respiratory viruses such as influenza^16^ and SARS-CoV-2 (ref. ^17^). We found IRF7 mRNA downregulated in pDCs following testosterone therapy, and IRF7 expression correlated with Hallmark IFNα transcripts in individual pDCs stimulated with R848 (Fig. 2j). The suppressor of cytokine (SOCS) family of regulators are triggered by JAK-STAT signalling downstream of several cytokine receptors, providing negative feedback regulation. SOCS3 dampens IFN-I responses during flu infection^18^ and we found SOCS3 (Fig. 2k) and, to some extent, SOCS1 (Extended Data Fig. 3c,d), upregulated in pDCs during in vivo testosterone therapy and inversely correlated with Hallmark IFNα and IRF7 following R848 stimulation (Extended Data Fig. 3e). These results offer further insights to testosterone-mediated attenuation of IFN-I responses in pDCs.

## Monocyte adaptation to testosterone therapy

**Fig. 3:**
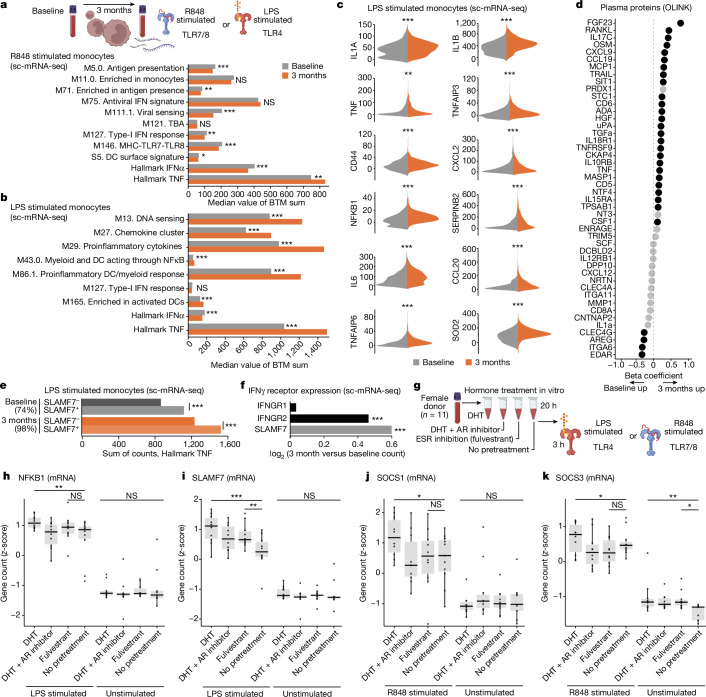
Monocyte responses following testosterone treatment. **a**, Median sum gene counts compared by two-sided, independent samples Student’s *t*-test, Bonferroni corrected *P* values for the indicated BTM in R848-stimulated (3 h) monocytes from baseline (*n* = 466) and after 3 months of testosterone (*n* = 851) treatment across two independent experiments. **b**, Median sum gene counts compared by two-sided, independent samples Student’s *t*-test with Bonferroni corrected *P* values for the indicated BTMs in LPS-stimulated (3 h) monocytes at baseline (*n* = 1,297) and 3 months (*n* = 1,050) from two independent experiments. **c**, log-transformed counts from sc-mRNA-seq of LPS-stimulated monocytes as in **b** after subtracting median expression of unstimulated cells at baseline (*n* = 1,297; grey) and 3 months (*n* = 1,050; orange) across two independent experiments. Twelve genes in the Hallmark TNF pathway are shown. **d**, Analysis of plasma proteins (Olink Target 96 inflammation and immune response panels) in samples from *n* = 20 participants at baseline and 3 months of testosterone in a single experiment. Black dots significantly different at 12 months over baseline (*P* < 0.05) by Kruskal–Wallis tests. **e**, sc-mRNA-seq and Hallmark TNF responses upon LPS stimulation (3 h) in SLAMF7 high versus low monocytes as in **b**. Fraction of SLAMF7^+^ monocytes at baseline (*n* = 1,297) and after 3 months of testosterone (*n* = 1,050) shown on top. Two-sided, independent samples and uncorrected Student’s *t*-test; ***P < 0.001. **f**, The log_2_ (fold change, 3 months versus baseline) gene counts for IFNGR1, IFNGR2 and SLAMF7 mRNA in monocytes (baseline, *n* = 1,297 and 3 months, *n* = 1,050). **g**, Blood from 11 healthy cis female participants incubated for 20 h with DHT with/without AR inhibitor (Enzalutamide) or ESR inhibitor/degrader (Fulvestrant) and then stimulated (3 h) by LPS or R848 and analysed for induced mRNA (*n* = 560) by Nanostring nCounter. **h**–**k**, *z*-score transformed mRNA counts of LPS-induced NFKB1 (**h**), SLAMF7 (**i**), R848-induced SOCS1 (**j**) and SOCS3 (**k**). One-sided, paired measurements, uncorrected Student’s *t*-tests, *P < 0.05; **P < 0.01; ***P < 0.001.

**Extended Data Fig. 4:**
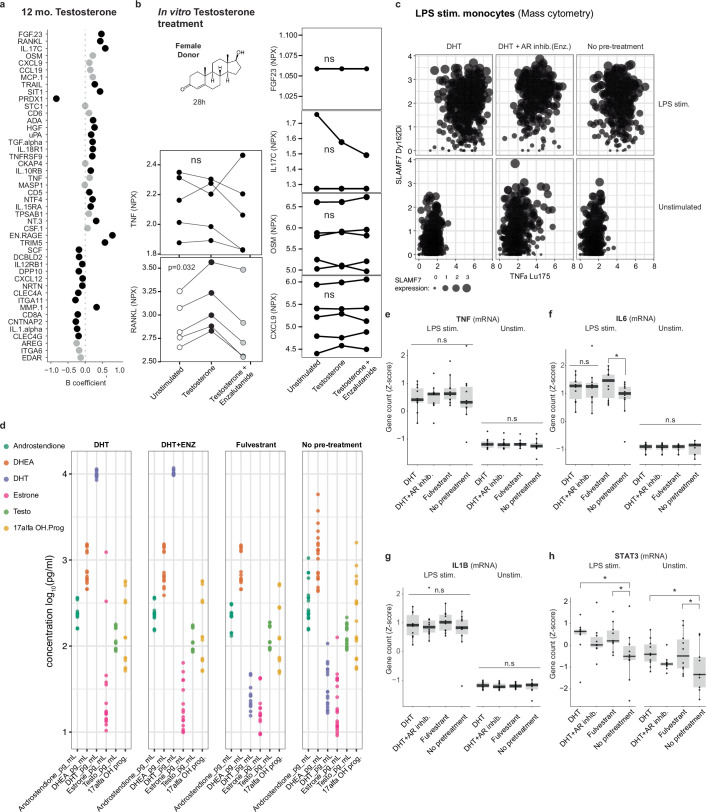
TNF family proteins and monocyte responses. **a**) Olink Target96^TM^ inflammation panel analyses of plasma samples from n = 20 subjects sampled 12 months after initiating Gender affirming testosterone therapy shown as mixed-effects modeling coefficients. **b**) In vitro testosterone treatment (28 h) of blood from a healthy female with/without AR antagonist Enzalutamide and analyses by Olink Target 96 Inflammation. Linear mixed effects analysis with treatment as fixed effect and subject as random effect and 5% FDR corrected p-values, n.s: non-significant **c**) Blood from a healthy female incubated (20 h) with dihydrotestosterone (DHT) with/without AR inhibitor (Enzalutamide) and stimulated by LPS (4 h) followed by Mass cytometry analysis of intracellular TNFα and SLAMF7 surface protein. Randomly down sampled (5%) of cells and visualized with dot size corresponding to SLAMF7 expression show TNFα^hi^ cells predominantly expressing high SLAMF7 upon DHT pre-treatment. A single representative experiment of three. **d**) Blood from eleven healthy cis females incubated (20 h) with dihydrotestosterone (DHT) with/without androgen receptor inhibitor (Enzalutamide) or oestradiol-receptor blocker and degrader (Fulvestrant). Resulting culture supernatants were analyzed for Androstenedione, DHEA, DHT, Estrone, Testosterone and 17a-Hydroxyprogesterone using GC-MS. **e**) Cultures were stimulated by LPS for 3 h and analyzed for mRNA-abundances (n = 560) by Nanostring nCounter. Z-score transformed mRNA (counts) of LPS induced TNF, **f**) IL6, **g**) IL1B, and **h**) STAT3. P-values: *p < 0.05, n.s: non-significant, by uncorrected pairwise t-tests. Boxplot centre = median, max whisker = Q3 + 1.5*IQR (IQR = values ranging from Q1-Q3), min whisker = Q1 – 1.5*IQR.

Monocytes were also analysed by scRNA-seq following R848 stimulation, showing attenuated Hallmark IFNα responses after 3 months of testosterone therapy (Fig. 3a). In contrast, Hallmark TNF responses upon R848 stimulation were potentiated in monocytes by testosterone therapy, indicating that the cross-regulation between IFN-I and TNF responses in blood mRNA-seq, is manifested in individual monocytes (Fig. 3a). Stimulation with lipopolysaccharide (LPS) for 3 h showed further potentiated monocyte TNF responses by testosterone therapy (Fig. 3b). Top genes involved in the Hallmark TNF response include IL-1, IL-6 and TNF but also NFκB pathway member NFKB1 were all induced more strongly by LPS stimulation after 3 months of testosterone in vivo (Fig. 3c). TNF family proteins such as TNF, RANKL, TNFSFR9 and TRAIL were elevated in plasma during testosterone therapy (Fig. 3d and Extended Data Fig. 4a). We treated blood from a healthy cisgender female participant with testosterone with or without the androgen receptor (AR) inhibitor enzalutamide and found RANKL was induced after 28 h in an AR-dependent manner (Extended Data Fig. 4b). We conclude that, in contrast to attenuated IFN-I responses by pDCs and monocytes, Hallmark TNF responses are potentiated during testosterone treatment, further underscoring the cross-regulation of IFN-I and TNF regulated by sex hormones. These findings are important for understanding immunological consequences of masculinizing GAHT in trans men, but perturbations to this regulatory axis can also explain cytokine storms and excess mortality in cis male patients over female patients with COVID-19 and other severe infections.

## SLAMF7 is induced by testosterone

A recently described surface receptor, SLAMF7, potentiates TNF responses through an autocrine loop in myeloid cells^19^. We found SLAMF7 upregulation in T cells and monocytes during testosterone therapy. Pretreatment of blood cells from a cisgender female participant with dihydrotestosterone (DHT)—a form of testosterone not convertible to oestradiol by aromatase—followed by LPS stimulation for 3 h, induced TNF production in monocytes at amounts (mean fluorescence intensity) that correlated with SLAMF7 expression (Extended Data Fig. 4c). scRNA-seq of monocytes from individuals undergoing GAHT and stimulated with LPS for 3 h ex vivo showed Hallmark TNF responses consistently more potent in SLAMF7^+^ than in SLAMF7^−^ monocytes, although both of these fractions were further enhanced by testosterone therapy (Fig. 3e). SLAMF7 is induced by IFNγ^19^ and we found one of its receptor genes, IFNGR2, upregulated in monocytes after testosterone treatment (Fig. 3f). These findings indicate that SLAMF7 upregulation could contribute to testosterone-mediated potentiation of TNF responses in monocytes.

## Relative effects of androgens and oestrogens

GAHT in individuals assigned female sex at birth decreases oestradiol concentrations rapidly and halts menstrual cycles. To investigate the relative contribution of increased testosterone and suppressed oestradiol on immune cell responses, we collected blood from 11 cisgender female participants of reproductive age and pretreated blood samples with testosterone, with and without the AR inhibitor enzalutamide as a control. Furthermore, as an alternative condition, we pretreated cells from the same donors with fulvestrant—a degrader of oestradiol-receptors (ESR) 1/2 (ESR1/2) to mimic the loss of ESR-mediated signals. We verified the expected hormone concentrations in each culture (Extended Data Fig. 4d). After 20 h of pretreatment, we stimulated cultures with LPS or R848 (3 h) and induced *n* = 560 mRNA transcripts (Fig. 3g). NFKB1 is a canonical LPS-induced gene, potentiated during GAHT, and also potentiated by 20 h pretreatment with DHT, but not Fulvestrant (Fig. 3h). AR inhibition prevented the effect, indicating a direct role of androgen signalling in potentiating NFKB1 upon LPS stimulation (Fig. 3h). Other Hallmark TNF pathway genes (IL-6, TNF and IL1B) were not induced significantly by either DHT or fulvestrant, indicating that further mechanisms or more time is required to mimic their induction in vivo (Extended Data Fig. 4e–g). SLAMF7 upregulation after LPS was stronger in either DHT- or Fulvestrant-pretreated cells, indicating a balance between androgens and oestrogens regulating this factor (Fig. 3i). Similarly STAT3 is an LPS response gene induced by either DHT or Fulvestrant, and this effect was visible even in unstimulated cultures without LPS (Extended Data Fig. 4h).

Negative regulators of IFN-I responses—SOCS1 and SOCS3—were upregulated by DHT pretreatment in an AR-dependent manner, but not by ESR inhibition (Fulvestrant) (Fig. 3j,k), verifying the direct effect of androgen signalling in suppressing IFN-I through these negative regulators in vivo during GAHT and in vitro.

## T cell adaptation during GAHT

**Extended Data Fig. 5:**
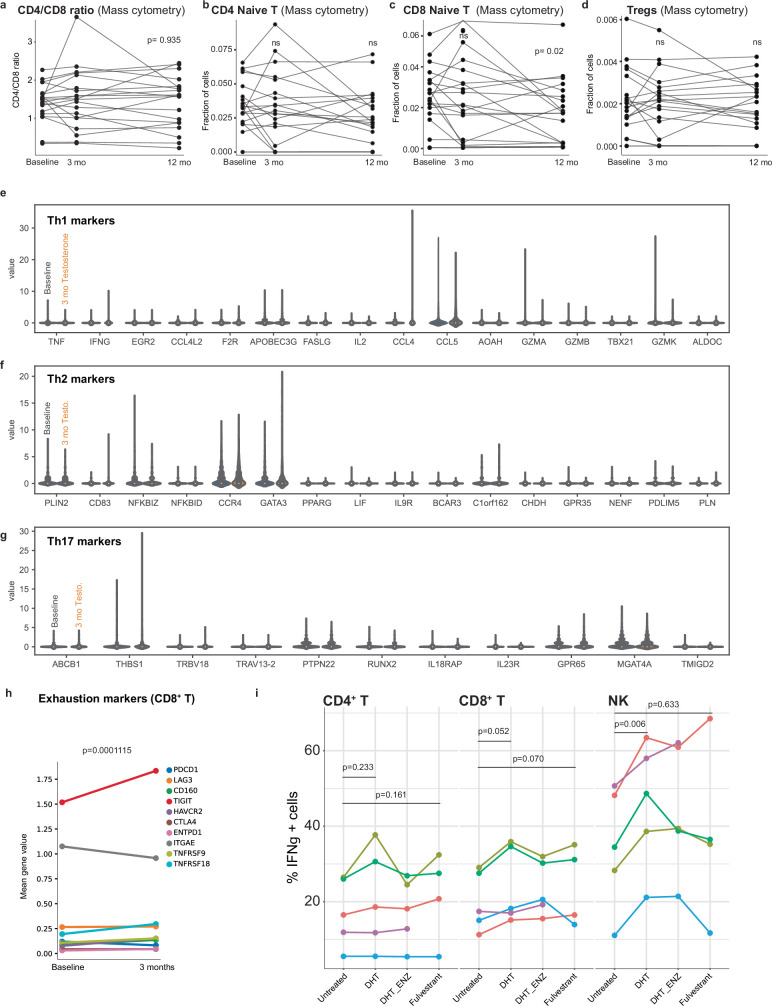
T cell adaptation to testosterone treatment. **a**) CD4/CD8 ratio, **b**) Naive CD4 + T cells, and **c**) Naive CD8 + T cell fractions before and during testosterone treatment. P-values from two-sided, paired and uncorrected t-tests. n.s = non-significant. **d**) Mass cytometry analyses of Treg frequency. P-values from two-sided, paired and uncorrected t-tests. n.s = non-significant. **e**) Single cell mRNA sequencing from PBMCs at baseline and after 3 months of testosterone in vivo selected on memory CD4 + T cells and transcripts related to Th1, **f**) Th2 and **g**) Th17 markers shown. **h**) Expression of the indicated T cell exhaustion markers for CD8 + T cells at baseline and 3 months following testosterone analyzed by single cell mRNA sequencing. P-value from 2-sided, uncorrected t-test indicating global module expression at baseline vs. 3 months. **i**) Five healthy female donors, pretreated with DHT, DHT + AR inhib. (Enzalutamide) or ESR inhibitor (Fulvestrant) followed by PMA/Ionomycin stimulation (4 h) and analyzed by intracellular IFNγ in CD4^+^ T, CD8^+^ T and NK cells analyzed by repeated measures ANOVA with Tukey’s Honest Significant Differences posthoc multiple hypothesis test.

CD4/CD8 T cell ratios were higher in female in than male participants^20,21^ but no decrease occurred during testosterone treatment (Extended Data Fig. 5a), indicating that genetic factors rather than steroids are responsible for this. In 1889, Calzolari reported enlarged thymi in castrated male rabbits^22^ and many subsequent studies confirmed this inhibitory effect on thymic output in mice^23^ and humans^24^ through AR-expressing thymic epithelial cells^25^. In our cohort, a slight reduction in naïve CD8^+^ T, but not CD4^+^ T cell proportions was seen after 12 months of testosterone treatment (Extended Data Fig. 5b,c). T_reg_ cells are more abundant in postpubertal male participants than in age-matched female participants^26^ but, during GAHT, frequencies were stable over 12 months (Extended Data Fig. 5d). Sex differences in CD4^+^ T cell polarization have been reported^27^, but in our sc-mRNA-seq data, no difference in T_H_1, T_H_2 or T_H_17 marker genes occurred during testosterone therapy (Extended Data Fig. 5e–g). T cell exhaustion in patients and mice with cancer has been linked to androgen signalling^28–30^. We found increased expression of a T cell exhaustion gene module in CD8^+^ T cells dominated by TIGIT mRNA upregulation (Extended Data Fig. 5h). These findings illustrate how specific immune system components, reportedly divergent between male and female participants, are regulated by chromosomal differences, whereas others change dynamically in response to changing sex hormones.

## Epigenetic induction of the NFκB pathway

**Fig. 4:**
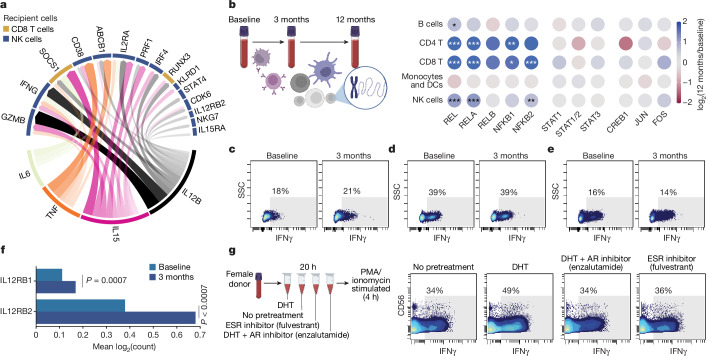
NFkB activation and IFNγ induction in NK cells following testosterone therapy. **a**, NicheNet analysis of monocytes from single-cell transcriptome data of LPS-stimulated PBMC comparing 3 months of in vivo testosterone treatment versus baseline. All target genes (top half of circle) are upregulated after testosterone treatment in vivo in NK cells and CD8^+^ T cells. Most explanatory genes in monocytes are shown in the lower half of the circle. Arrow width and density correspond to strength of inferred relationship. **b**, Blood T cells analysed for TF binding site chromatin accessibility as log-fold enrichment at 12 months versus baseline for a given TF with indicated cell populations using sc-ATAC-sequencing of PBMC (*n* = 12,773) from three participants sampled before and during testosterone treatment. Cells were assigned to indicated cell populations on the basis of gene activity for canonical marker genes. Adjusted *P* values: *P < 0.05, **P < 0.01, ***P < 0.001. **c**–**e**, PBMCs obtained at baseline or after 3 months of testosterone treatment were simulated with PMA/ionomycin for 4 h in vitro and intracellular IFNγ production in NK cells (**c**), CD8^+^ T cells (**d**) and CD4^+^ T cells (**e**) was analysed using flow cytometry. Numbers indicate percentage IFNγ^+^ cells. **f**, Expression of IL12RB1 and IL12RB2 mRNA in NK cells at baseline and after 3 months of in vivo testosterone treatment by sc-mRNA-seq. Two-sided, independent samples and uncorrected Student’s *t*-test; ***P < 0.001. **g**, Blood from one healthy cisgender female participant was incubated for 20 h with DHT with/without Enzalutamide or Fulvestrant followed by stimulation with PMA/ionomycin for 4 h, staining for intracellular cytokines and analysis by mass cytometry. Manually gated NK cells are shown and the fraction of IFNγ^+^ cells was determined on the basis of staining controls as indicated.

As testosterone modulated monocyte function strongly during masculinizing GAHT, we performed NicheNet analyses to infer downstream consequences on other immune cell populations. We found monocyte-derived IL-6, TNF and IL-15 were upregulated by testosterone, and monocyte-released IL-12B as candidate genes to best explain several upregulated transcripts measured in NK and CD8^+^ T cells during GAHT (Fig. 4a). The IFN-I regulator SOCS1 and the transcription factor (TF) RUNX3—important for maintaining cytotoxic function of CD8^+^ T cells^31^—can be explained by increased monocyte-derived IL-12B and IL-15. In NK cells, induced transcripts associated with cytotoxic function (GZMB, PRF1 and NKG7) were also explained by upregulated IL-15 and IL-12B (Fig. 4a). Furthermore, upregulated IFNγ mRNA in NK cells was predicted as a downstream consequence of monocyte-derived IL-6, IL-15, IL-12B and TNF (Fig. 4a).

To investigate whether testosterone therapy would induce epigenetic adaptations in individual immune cells, we performed single-cell ATAC sequencing (sc-ATAC-seq) of (*n* = 12,773) PBMCs from three participants sampled at baseline and after 3 and 12 months of testosterone treatment (Fig. 4b). Given the altered IFN-I/TNF cross-regulation, we focussed on chromatin accessibility changes at JAK-STAT, AP-1, NFκB and MAPK TF binding sites. We found increased TF activity for all canonical NFκB binding sites, but not RELB (non-canonical NFκB) in T cells and NK cells, but not in B cells or myeloid/DCs following 12 months of testosterone treatment (Fig. 4b). No significant changes were observed for STAT1, STAT2, STAT3, AP-1 (Fig. 4b) or MAPK binding sites (data not shown), indicating that the canonical NFκB pathway is induced epigenetically in T/NK cells following testosterone treatment, possibly as a consequence of elevated TNF responses by myeloid cells, with broad functional implications for T/NK cell function and proliferation^32^.

## Potentiation of IFNγ responses by NK

To further assess functional consequences of GAHT on lymphocytes, we stimulated PBMCs at baseline and following 3 months treatment with phorbol 12-myristate 13-acetate (PMA)/ionomycin and assessed intracellular IFNγ responses by flow cytometry. We found stronger IFNγ responses in NK cells following 3 months of testosterone treatment (Fig. 4c), whereas CD8^+^ (Fig. 4d) and CD4^+^ T cell responses were unchanged (Fig. 4e). IL12RB1 and IL12RB2 mRNA in individual NK cells were induced during GAHT. Collectively, these findings support the view that NK cell function is potentiated during GAHT as a consequence of induced IL-12 responses by monocytes following testosterone treatment (Fig. 4f).

We also aimed to distinguish relative effects of added testosterone through AR-signalling from the loss of oestradiol-mediated signals during GAHT. To this end, blood from five healthy cisgender female participants was preincubated with DHT, DHT + AR inhibitor (enzalutamide) or ESR inhibitor (fulvestrant) alone (20 h), before stimulation with PMA/ionomycin (4 h) (Fig. 4g). Intracellular IFNγ was measured by mass cytometry and we found that pretreatment with DHT, but not loss of ESR signalling (fulvestrant), potentiated IFNγ responses by CD56^dim^ NK cells, but not T cells (Fig. 4g and Extended Data Fig. 5i). These findings indicate a loop of potentiated IFNγ responses by NK cells and IFNγ-mediated upregulation of SLAMF7 on monocytes associated with potentiated TNF responses, triggering epigenetic activation of NFκB-regulated genes in T/NK cells and further enhancing IFNγ production by NK cells during testosterone therapy.

## Corroborating findings in cisgender cohorts

**Fig. 5:**
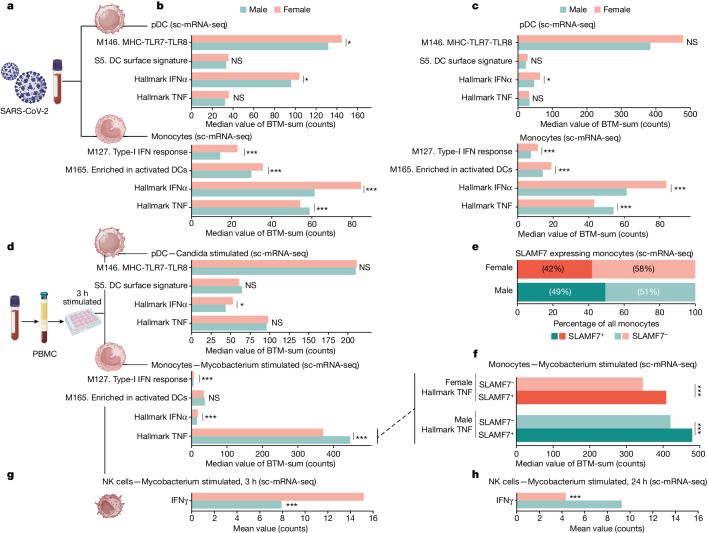
Sex-divergent responses confirmed in public datasets. **a**, Male and female patients infected with SARS-CoV-2. **b**, sc-mRNA-seq data from patients infected with SARS-CoV-2 selected for pDCs (*n* = 144 (male, 103; female, 41)) and monocytes (*n* = 33,887 (male, 18,262; female, 15,625)) and gene count sum for the indicated BTM. Two-sided, independent samples and uncorrected Student’s *t*-test: *P < 0.05; **P < 0.01; ***P < 0.001. **c**, PBMC data from SARS-CoV-2 infected patients analysed by sc-mRNA-seq and divided into pDCs (*n* = 21 (male, 10; female,11)) and monocytes (*n* = 4,521 (male, 2,672; female, 1,849)). Two-sided, independent samples and uncorrected Student’s *t*-test: *P < 0.05; **P < 0.01; ***P < 0.001. **d**, PBMCs from healthy male and female participants stimulated in vitro (3 h) and analysed by sc-mRNA-seq. pDCs (*n* = 262 (male, 162; female, 100)) were selected from cells stimulated with *C. albicans*, and monocytes (*n* = 12,961 (male, 6,652; female, 6,309)) were selected from cells stimulated with mTB. Two-sided, independent samples and uncorrected Student’s *t*-test: *P < 0.05; **P < 0.01; ***P < 0.001. **e**,**f**, Monocytes in **d** subdivided according to SLAMF7 expression (**e**), and Hallmark TNF gene count following mTB stimulation in vitro for SLAMF7^+^ and SLAMF7^−^ monocytes (**f**). Two-sided, independent samples and uncorrected Student’s *t*-test: *P < 0.05; **P < 0.01; ***P < 0.001. **g**,**h**, Single NK cell transcriptome analyses following in vitro exposure to mTB for 3 h (**g**) and 24 h (**h**) and mean mRNA count for IFNγ are shown. Two-sided, independent samples and uncorrected Student’s *t*-test: *P < 0.05; **P < 0.01; ***P < 0.001.

To investigate whether observations made in individuals undergoing masculinizing GAHT could also explain divergent immune responses in cisgender individuals, we analysed several sc-mRNA-seq datasets of immune cells from male and female participants (Fig. 5a). SARS-CoV-2 triggers sex-divergent immune responses and we found cross-regulated IFN-I and TNF responses in pDCs and monocytes, as shown by significantly higher Hallmark TNF responses in male monocytes and reduced IFN-I responses in two cohorts of adults less than 50 years of age (Fig. 5b,c)^33,34^. In a separate sc-mRNA-seq dataset^35^ of PBMCs from healthy volunteers stimulated in vitro with *Candida albicans* or *Mycobacterium tuberculosis* (mTB), greater IFN-I responses were seen in female pDCs and monocytes, while Hallmark TNF responses were higher in male monocytes (Fig. 5d), further supporting sex hormone-mediated regulation of the IFN-I/TNF axis as an explanation for divergent responses by cis male and female participants across several cohorts.

Using the same dataset^35^, we found higher frequencies of SLAMF7^+^ monocytes in male participants (Fig. 5e), and these SLAMF7^+^ monocytes produced stronger Hallmark TNF responses following mTB stimulation as compared with SLAMF7^−^ monocytes, indicating that this feature described during masculinizing GAHT also contributes to sex-divergent responses in cisgender individuals (Fig. 5f). In PBMCs stimulated with mTB, sex-divergent NK cells responses were also found. After 3 h of mTB stimulation, the IFNγ mRNA response was stronger in female NK cells, probably reflecting differences in sensing mechanisms and initial IFN-I/II responses in female cells (Fig. 5g). In contrast, after 24 h of mTB in vitro, secondary signals, such as monocyte-derived IL-6, TNF, IL-15 and IL-12B, are likely to influence, and in this case female NK cells have markedly reduced IFNγ mRNA, whereas male NK cells sustain a high IFNγ response transcriptionally (Fig. 5h). This difference in response dynamics points towards cell–cell interactions and regulatory mechanisms divergent between sexes and corroborate our predictions from individuals undergoing GAHT. Testosterone-mediated potentiation of a monocyte–NK cell axis resulting in upregulated TNF and IFNγ can offer explanations for previously reported sex differences in mTB disease course^36^. Collectively, our results highlight previously unrecognized immunomodulatory effects of sex hormones in humans, calibrating an IFN-I/TNF axis across several immune cell populations with implications for understanding sex-divergent immune responses to infections, vaccines and autoantigens in immune-mediated diseases.

## Discussion

Sex hormones, in contrast to sex chromosomes, offer an opportunity for dynamic regulation of the human immune system in relation to changing needs over the course of life, and even during the course of the menstrual cycle in women of reproductive age. Sex chromosomes on the other hand, encode immunological differences between male and female individuals selected for throughout evolution^37^. The inactive X and active Y chromosomes can broadly modulate autosomal gene expression^8^, and gain-of-function mutations in the X-encoded gene TLR7 can trigger monogenic lupus-like disease with elevated IFN-I responses^38^.

GAHT enables the acquisition of secondary sex characteristics aligned with gender identity in transgender individuals but the immunological impacts and risks of immune-mediated and infectious diseases upon sex hormone therapy is unknown. Here we describe several layers of immunological adaptations in trans men, assigned female sex at birth and undergoing masculinizing testosterone treatment.

We find that testosterone increase and the resulting oestradiol suppression, alters a cross-regulation axis involving IFN-I and TNF. Similar cross-regulation has been described in vitro^39^ and in vivo in healthy volunteers^40^, and in patients with female-dominated autoimmune lupus^41^, but its regulation by sex hormones is not previously known. Curiously, around 15% of patients treated with anti-TNF develop a lupus-like syndrome with autoantibodies against double-stranded RNA and elevated IFN-induced gene signatures^42^. Detailed studies of IFN-I and TNF cross-regulation have been performed in osteoclasts and show that TNF, RANKL and CSF1 induce NFκB-regulated genes, while inhibiting IFN-I (ref. ^43^). Conversely, IFN-I signalling through IFNAR1 limits this NFκB and AP-1 activation^43^. During GAHT, we find that plasma RANKL, CSF1 and TNF are all induced, NFκB TF binding sites exhibit more chromatin accessibility and Hallmark TNF responses are enhanced in an AR-dependent manner, indicating that IFN-I/TNF cross-regulation occurs at the system level and is calibrated by sex hormones in vivo.

The question arises as to whether observed changes are associated directly with testosterone treatment or occur indirectly due to reduced oestradiol signals. To test this question, we designed an in vitro system in which blood from 11 cis female donors was pretreated with either DHT (in the presence or absence of the AR inhibitor enzalutamide) or the ESR inhibitor fulvestrant only. Results showed that NFκB-mediated responses to LPS stimulation were potentiated by DHT alone through AR and not by loss of ESR-mediated signals. Inhibitors of IFN-I responses—SOCS1 and SOCS3—were also induced by DHT alone, indicating direct effects of androgens in suppressing IFN-I responses. It is conceivable that loss of oestradiol-mediated signals can also contribute to attenuated IFN-I responses through other mechanisms not investigated herein. In support of this, Seillet et al previously showed that oestradiol supplementation potentiates IFNα responses in postmenopausal women^44^ and Griesbeck et al. reported that ESR signalling in mice potentiates IFNα responses in pDCs through IRF5 induction^45^. A case of a trans woman developing lupus during oestradiol therapy^46^ points towards direct potentiating effects of oestradiol on IFN-I responses.

Male patients are at increased risk of severe disease following SARS-CoV-2 infection due to less efficient IFN-I responses and excessive inflammation mediated by IL-6, IFNγ, TNF and IL-1/18 (ref. ^1^). This could be explained in part by the testosterone-mediated potentiation of proinflammatory responses through IFNγ, SLAMF7 and TNF reported herein. Female patients with polycystic ovary syndrome have elevated testosterone concentrations, elevated plasma TNF^47^ and are at greater risk of severe COVID-19 as compared with age-matched female control participants without polycystic ovary syndrome^48^. Not much is known about the severity of SARS-CoV-2 infections in individuals undergoing GAHT but one single-centre study found higher rates of symptomatic COVID-19 in trans men on testosterone therapy as compared with trans women on oestradiol^49^. Whether this reflects immune response differences and more symptomatic disease or differences in risks of contracting the virus through modulated ACE2 and or TMPRS2 expression remains to be determined.

Recent developments in immune-oncology have shown important roles for AR signalling in regulating T cell responses to tumours and limiting the efficacy of checkpoint blockade through T cell exhaustion^28–30^. We find upregulation of TIGIT—a marker of T cell exhaustion during masculinizing GAHT—in vivo, but we also find induction of TNF responses and IFNγ responses by NK cells, which seems at odds with reported impairments of T_H_1 responses in male prostate cancer patients. However, analyses of chronic infection models (human immunodeficiency virus or lymphocytic choriomeningitis virus) have indicated TNF signalling in T cells as another important regulator of T cell exhaustion during chronic antigen stimulation^50^. It is tempting to speculate that the testosterone-driven induction of TNF described herein could be another driver of T cell exhaustion in cancer patients and a possible target for combination therapies in the future.

The main drawback of this study is the limited cohort size and the challenges in studying mechanisms of hormone-induced changes in human participants. Studies of model organisms are warranted to clarify mechanisms in vivo but, unlike higher order primates, laboratory mice do not menstruate^51^ and also differ in their sex hormone regulation, affecting trade-off mechanisms in relation to changing needs. Performing in vitro experiments using human cells as described herein offers some mechanistic understanding but is limited in that such experiments are disconnected from physiological mechanisms of regulation in vivo through the nervous system or other endocrine pathways of importance.

**Extended Data Fig. 6:**
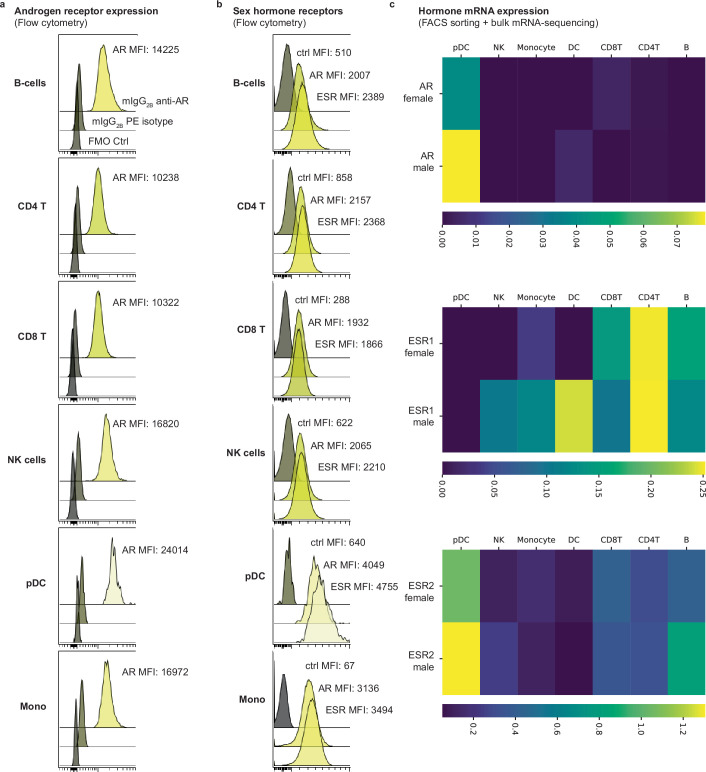
Sex-hormone receptor expression. **a**) Flow cytometry analysis of intracellular staining of the androgen receptor, AR in the indicated cell populations. Staining control (fluorescence minus one, FMO) and mouse anti-human IgB_2B_ PE isotype control in grey. Mean Fluorescence Intensity, MFI is indicated. **b**) Flow cytometry analysis of AR and pan-ESR in the indicated cell populations. Staining control (fluorescence minus one, FMO) in grey. **c**) PBMCs from healthy men and women sorted based on canonical surface markers and subject to bulk mRNA-sequencing and expression nTPM (normalized Transcripts per million bases) for the indicated sex hormone receptor mRNA in three female and three male donors combined.

Another challenge in human studies is separating the direct effects of gonadal steroids on a particular cell type from secondary effects mediated through cell–cell communication in vivo. Expression of the AR varies across cell populations, with maximal protein amounts found in pDCs followed by monocytes, while lymphocyte populations express lower amounts of AR protein (Extended Data Fig. 6a). The two ESRs show variable expression across immune cell types but highest protein expression in pDCs (Extended Data Fig. 6b). Analyses of mRNA from cell populations derived from fluorescence-activated cell sorting (FACS) show a similar pattern, with highest expression in pDCs in both male and female cells (Extended Data Fig. 6c) and varied AR/ESR expression amounts across cell types, which offers another layer of complexity to the regulation of immunity by these hormones.

Life history theory provides a framework for understanding allocation of limited resources between critical traits such as reproduction, growth and maintenance, in which immunity is a key component^52^. Male investment into reproduction is much lower than that of females, and males allocate more resources into muscle growth and immune function and testosterone is a key regulator of such resource allocation in vertebrates^53^. Metcalf and Graham modelled trade-offs between sex-divergent recognition of pathogens (female superior) and pathogen-killing processes (male superior) and found that sexual maturation and changes in reproductive investments during the life course explains these observed divergent immune processes^54^. Our results corroborate this and adds mechanisms of hormone-mediated regulation of immunity in response to changing needs during the life course.

Evolutionary pressures from pathogens have shaped human immune systems and the risk of vertical transmission is a selective pressure unique to pregnant females^55^. It is thus tempting to speculate that potentiated IFN-I induced by elevated oestradiol amounts in pregnancy is selectively favoured to limit such vertical transmission of viruses. This also corroborates clinical observations of increased lupus flares mediated by IFN-I during pregnancy^56^. TNF is critical for the defence against *Mycobacteria*, *Staphylococci* and other common bacteria, but elevated TNF is also associated with failure of implantation^57^, pregnancy loss^58^ and preeclampsia^59^. Evolutionary pressures to suppress TNF while potentiating IFN-I during the second half of the menstrual cycle and following implantation in pregnant people could increase the likelihood of reproductive success. Conversely, in male individuals, we speculate that increased investment into muscular growth could explain testosterone-mediated potentiation of TNF and suppression of IFN-I. Testosterone is induced following acute exercise and transient TNF responses by tissue macrophages is important for muscle regeneration and growth^56^. Chronically elevated TNF leads to muscle wasting. IFN-I directly inhibits myoblasts, leading to muscle weakening and wasting as seen in patients with autoimmune dermatomyositis^60^. To this end, it is conceivable that hormone control of the TNF/IFN-I axis reflects these different investments in reproduction and muscle growth, respectively, as predicted by life history theory.

Understanding the mechanisms by which gonadal steroids modulate immunity in individuals undergoing masculinizing GAHT are important to ensure the health and wellbeing of trans men and avoid long-term adverse outcomes such as severe infection and inflammatory disorders. These mechanisms can also help explain the divergent immune responses in cis men and women that are regulated dynamically by sex hormones in relation to ever changing needs during the human life course.

## Methods

### Inclusion and ethics

Twenty-three adult individuals who were assigned female sex at birth and who were undergoing masculinizing gender-affirming treatment were enroled at specialist centres for transgender medicine in Stockholm, Uppsala, Linköping and Umeå in Sweden between 2016 and 2023. The study was approved by the Swedish Ethical Review Authority (2016/1422-31/1). Informed consent was obtained from all individuals. Only individuals who had not previously received testosterone treatment and who had normal sex hormone concentrations were included. Additionally, individuals with autoimmune diseases, immunodeficiencies or signs of continuing infection/inflammation were excluded from the study.

### Testosterone therapy

Venous blood samples were collected at three timepoints: baseline, 3 months and 12 months following testosterone injections (Testosterone Undecaonate, Nebido administered once every 12 weeks). All patients received 1,000 mg of Nebido except for four participants who received reduced doses of 750 mg due to low body mass indices or haematocrit values. The baseline sample was collected within the 2 weeks before the start of testosterone treatment.

### Measurement of serum sex hormones

Serum concentrations of sex hormones were analysed using liquid chromatography with tandem mass spectrometry assays at Gothenburg University as described previously^61^. The same method was used to analyse sex hormones from resulting culture supernatants as shown in Extended Data Fig. 4d. Briefly, calibrator stock solutions were prepared for all sex hormones and each internal standard stock solution was made separately using ^13^C_3_-labelled versions of each steroid, except for dehydroepiandrosterone, which was labelled with d6. Steroid hormones were analysed using a two-dimensional liquid chromatography system consisting of an Acquity ultra-performance liquid chromatography system and a TQ-XS triple quadrupole mass spectrometer from Waters. The lower limit of detection (LLOD) was defined as the lowest peak with a signal more than three times the noise level. The lower limit of quantification (LLOQ) was defined as the lowest peak that was reproducible with a coefficient of variation of less than 20% and an accuracy of 80% to 120%. To circumvent problems with endogenous steroid amounts, the determinations of LLOD and LLOQ were performed in human serum pools with isotope-labelled steroids spiked at four different concentrations.

The amounts of bioavailable testosterone were calculated according to the formulae below^62^:

$${\rm{B}}{\rm{i}}{\rm{o}}{\rm{a}}{\rm{v}}{\rm{a}}{\rm{i}}{\rm{l}}{\rm{a}}{\rm{b}}{\rm{l}}{\rm{e}}\,{\rm{t}}{\rm{e}}{\rm{s}}{\rm{t}}{\rm{o}}{\rm{s}}{\rm{t}}{\rm{e}}{\rm{r}}{\rm{o}}{\rm{n}}{\rm{e}}=({r}_{3}\times 0.5-{r}_{4})\times {r}_{5}/{r}_{6}$$

$${r}_{1}=(1+{\rm{r}}{\rm{K}}{\rm{b}},{\rm{A}}{\rm{L}}{\rm{B}}\times {\rm{P}}-{\rm{A}}{\rm{L}}{\rm{B}}+{\rm{r}}{\rm{K}}{\rm{b}},{\rm{S}}{\rm{H}}{\rm{B}}{\rm{G}}\times ({\rm{S}}-{\rm{S}}{\rm{H}}{\rm{B}}{\rm{G}}-{\rm{S}}-{\rm{T}}{\rm{E}}{\rm{S}}{\rm{T}}))\times 2$$

$${r}_{2}=4\times {\rm{r}}{\rm{K}}{\rm{b}},{\rm{S}}{\rm{H}}{\rm{B}}{\rm{G}}\times (1+{\rm{r}}{\rm{K}}{\rm{b}},{\rm{A}}{\rm{L}}{\rm{B}}\times {\rm{P}}-{\rm{A}}{\rm{L}}{\rm{B}})\times (-{\rm{S}}-{\rm{T}}{\rm{E}}{\rm{S}}{\rm{T}})$$

$${r}_{3}={{\rm{r}}}_{1}-{{\rm{r}}}_{2}$$

$${r}_{4}=(1+({\rm{r}}{\rm{K}}{\rm{b}},{\rm{A}}{\rm{L}}{\rm{B}}\times {\rm{P}}-{\rm{A}}{\rm{L}}{\rm{B}})+{\rm{r}}{\rm{K}}{\rm{b}},{\rm{S}}{\rm{H}}{\rm{B}}{\rm{G}}\times ({\rm{S}}-{\rm{S}}{\rm{H}}{\rm{B}}{\rm{G}}-{\rm{S}}-{\rm{T}}{\rm{E}}{\rm{S}}{\rm{T}}))$$

$${r}_{5}=(1+({\rm{r}}{\rm{K}}{\rm{b}},{\rm{A}}{\rm{L}}{\rm{B}}\times {\rm{P}}-{\rm{A}}{\rm{L}}{\rm{B}})$$

$${r}_{6}=2\times {\rm{r}}{\rm{K}}{\rm{b}},{\rm{S}}{\rm{H}}{\rm{B}}{\rm{G}}\times (1+{\rm{r}}{\rm{K}}{\rm{b}},{\rm{A}}{\rm{L}}{\rm{B}}\times {\rm{P}}-{\rm{A}}{\rm{L}}{\rm{B}})$$

in which rKb,ALB is the binding constant (0.601) for testosterone (TEST) to albumin (ALB), rKb,SHBG is the binding constant (1.0) for testosterone to sex hormone-binding globulin (SHBG) and P − ALB is a fixed value of 42.

### Sample processing

A 4 ml sample of blood was drawn in EDTA-containing sterile vacutainer tubes from each participant in the sex reassignment therapy cohort and prepared as follows: 0.5 ml of blood was mixed with an equal amount of whole blood stabilizer^63^ (Cytodelics AB), incubated for 10 min at ambient temperature and stored at −80 °C. A 1 ml aliquot of blood was mixed with PAXgene solution (BD Biosciences), incubated for 2 h at ambient temperature and stored at −80 °C. The remaining blood was centrifuged at 4 °C and 1,200*g* for 10 min, after which plasma was collected and stored at −80 °C. The leftover blood after plasma removal was mixed equally with PBS and layered over Lymphoprep (STEMCELL Technologies) for PBMC isolation by density gradient centrifugation following the manufacturer’s protocol. Cells were washed, counted and cryopreserved in a solution of 90% FBS (Sigma-Aldrich) mixed with 10% dimethylsulfoxide (DMSO; Sigma-Aldrich), initially stored at −80 °C overnight and then transferred to −150 °C for future use.

### Bulk RNA-seq of whole blood samples

To analyse changes in gene expression, we performed RNA-seq using RNA extracted from PAXgene blood samples. The RNA samples were prepared using a QIAcube with the PAXgene Blood RNA Kit (Qiagen). Before cDNA library preparation, the quality of the RNA was assessed by determining the RNA integrity number using the Agilent 2100 Bioanalyzer with the RNA 6000 Pico Kit. The RNA concentration was measured using the Qubit Fluorometer with the Qubit dsDNA HS Kit (ThermoFisher Scientific).

For final sequencing and cDNA library preparation, an Advanta RNA-Seq XT NGS Library Preparation Kit was used with the Juno system (Standard BioTools Inc.). We performed Bulk RNA-seq on a NovaSeq 6000 instrument using one flow cell SP-200 (Illumina) with paired-end reads and a read length 2 × 100.

### Data analysis of bulk mRNA-seq data

Bulk RNA-seq results from 59 samples from 20 individuals undergoing testosterone treatment were preprocessed with Kallisto^64^. Quality control was provided by the National Genomics Infrastructure at Science for Life Laboratory, Stockholm, Sweden. To generate abundance estimates for all samples, the Kallisto program (v.0.46.2) was used to quantify abundances of transcript sequences in FASTA format using the Ensembl transcriptome Homo_sapiens.GRCh38.cdna.all.index (https://ftp.ensembl.org/pub/release-109/fasta/homo_sapiens/cdna/) for the Kallisto index. The Kallisto outputs were then imported into R using the tximport package, and the effect of ‘visit’ on whole blood mRNA expression was assessed using DESeq2 (ref. ^65^) while accounting for interindividual variability and age effects. Before assessing differential gene expression, genes with fewer than 100 reads across samples were filtered out, as well as genes that did not have a normalized count of ten in at least one-fourth of the samples. The results from the differential gene expression analysis were used for gene set enrichment analysis of Hallmark pathways using clusterProfiler^66^.

### scRNA-seq experiments

Cryopreserved PBMCs obtained at baseline and after 3 months of testosterone treatment were thawed in thawing medium (RPMI 1640 HyClone supplemented with 10% FBS, 1% penicillin-streptomycin and Benzonase-nuclease (Sigma-Aldrich)). Cells were counted using a Cellaca MX (Nexcelom), plated and incubated for 1 h at 37 °C and 5% CO_2_ to rest. Samples were then either left untreated or stimulated ex vivo with LPS (100 ng ml^−1^) or R848 (1 μg ml^−1^) for 4 h. After stimulation, the cells were collected, and supernatants were stored for later analysis by SIMOA (Quanterix)^67^.

Viability and cell counts were assessed after resuspending collected cells in PBS with 0.04% BSA (ThermoFisher Scientific). The cells were then prepared for scRNA-seq using the 10x Genomics 3′ v.3.1 (dual index) kit according to the manufacturer’s instructions (catalogue no. CG000315 Rev B) on a Chromium Controller. Approximately 1 × 10^4^ cells from each condition were loaded onto separate wells of a 10x Genomics chip and the Chromium Controller was used to create GEM emulsions. The target recovery was 6,000–7,000 cells per condition. The libraries were sequenced on an Illumina NovaSeq 6000 platform, using paired- end reads (configuration 28 × 10 × 10 × 90) with 20,000 reads per cell.

### scRNA-seq data analyses

CellRanger with default parameters was used to process FASTQ-files and align sequencing reads from 10x Genomics 3′ HT v.3. and 3′ GE towards the human genome. Cells were further filtered using a bimodal distribution-based approach, excluding those with read counts below (considered low quality) or above (considered technical artifacts) cut-off thresholds. The cut-off thresholds for each sample were chosen on the basis of distribution shape of read counts to retain biologically relevant cells and to eliminate technical artifacts. Cells with mitochondrial gene expression above 10% were also filtered out. All scRNA-seq data were preprocessed in Python using Scanpy v.1.9.1. For each sample, normalization by counts per cell (target sum = 1 × 10^4^) and feature scaling were applied to the CellRanger outputs for each sample, followed by linear dimensionality reduction using PCA and uniform manifold approximation and projection (on top 2000 variable genes), nearest neighbours (*n* = 10) computation and identification of clusters (res = 1). Clusters were annotated on the basis of canonical marker genes. BTMs^68^ were used to compare transcriptional patterns before and during testosterone treatment and in response to stimulation.

### NicheNet analyses

The NicheNet analysis and circus plots were created following the standard workflow available from NicheNet^69^ and circlize^70^. Specifically, differentially expressed genes between samples from baseline and after 3 months of testosterone treatment were identified using Seurat’s (v.4.3.0) built in function FindMarkers and filtered with an adjusted *P* value of less than 0.05 and an absolute value for the average fold change of at least 0.15. Ligand activities were calculated, and the top upstream ligands that could explain the observed target gene expression changes were selected. The ligand–target links were filtered on the basis of their weights (strength of the ligand–target relationship), with links belonging to the lowest 66% of scores being removed. The circos plot blocks were coloured according to a gene’s target cell, inferred as the cell type with the highest mean-value change between the two visits. The widths of the blocks indicate the potential of each receptor to be influenced by all shown ligands, with some interactions not visible due to the cut-off weight threshold. The transparency of the arrows indicates the regulatory potential of a ligand–target interaction (the more transparent, the weaker the regulatory potential).

### sc-ATAC-seq and data analysis

sc-ATAC-seq experiments were conducted on the 10x Chromium platform, following a previously described protocol^71^. Briefly, cells were washed with PBS containing 0.04% BSA and nuclei subjected to isolation as per the manufacturer’s instructions. After counting, approximately 10,000 nuclei were used for tagmentation. The tagmented nuclei were then loaded for capture using the 10x Chromium controller. Following gel emulsion generation, we carried out linear amplification and DNA purification according to the manufacturer’s protocol. The resulting DNA was used for library construction, following the guidelines provided on the manufacturer’s website. The libraries were quantified using an Agilent Bioanalyzer and sequenced on an Illumina NovaSeq S4 sequencer, with the following setup: 50 bp read 1N, 8 bp i7 index, 16 bp i5 index and 50 bp read 2N. In this setup, 1N and 2N refer to the DNA insert sequencing, while i5 and i7 sequencing identify the individual barcodes of single cells.

The 10X Genomics cellranger pipeline (cellranger-atac mkfastq, count and aggr) was followed for 10x sc-ATAC-seq analysis. Cellranger aggr outputs were used for downstream analysis in R using the Signac package. We performed quality control using Signac’s default settings for transcriptional start site enrichment score, nucleosome banding pattern, sequencing depth and complexity, and fraction of fragments in peaks. The ratio of reads in genomic blacklist regions was calculated using the FractionCountsInRegion function with the blacklist for hg38. After quality control, a total of 143,624 peaks (features) across 12,773 cells remained for further analysis. The number of cells per sample varied between 636 and 4,632 for the eight total samples analysed. We applied frequency-inverse document frequency normalization, followed by feature selection and dimensionality reduction using singular value decomposition on the frequency-inverse document frequency matrix. We performed uniform manifold approximation and projection dimensionality reduction^72^ on the first 30 latent semantic indexing components, with latent semantic indexing components capturing technical variation excluded from further analysis. K-nearest neighbour graph construction and clustering were done using the smart local moving algorithm, resulting in the identification of 21 unique clusters. Gene activities were used for cluster annotation, with gene activities determined using the GeneActivity function followed by log normalization. Five main immune clusters were identified and used for further analyses. TF motif analysis was conducted by identifying overrepresented motifs in a set of differentially accessible peaks between pre- and post-testosterone therapy (3 or 12 months) for all the five immune subsets using hypergeometric tests and *P* values corrected for several hypotheses (Benjamini–Hochberg).

### Immune cell profiling by mass cytometry

Blood samples were mixed with a stabilizer^63^ (Whole blood processing kit component; Cytodelics AB) within the first hour post blood-draw and cryopreserved according to the manufacturer’s recommendations. Samples were then thawed, fixed and lysed using Lysis and Wash buffers (Whole blood processing kit; Cytodelics AB). After fixation/lysis, 1–2 × 10^6^ cells per sample were plated and cryopreserved using CRYO#20 (Cytodelics). For staining, cells were thawed at 37 °C, barcoded using an automated liquid handling robotic system (Agilent Technologies)^73^ using the Cell-ID 20-plex Barcoding kit (Standard BioTools Inc.) as per the manufacturer’s recommendations and stained batch-wise after pooling. Cells were washed using cell staining buffer (CSB) (Standard BioTools Inc.), FcR blocked using an in-house-prepared blocking solution for 12 min at ambient temperature then stained using a cocktail of metal-conjugated antibodies targeting surface antigens (Broad extended panel) and incubated for 30 min at 4 °C. Cells were washed twice with CSB and fixed overnight using 2% formaldehyde in PBS (VWR international). The panel of antibodies used is listed in Supplementary Table 1.

For cells from whole blood pretreated and stimulated in vitro, we performed intracellular staining. Cells were first stained with a cocktail of antibodies targeting surface antigens (Supplementary Table 2) and then washed twice with CSB, fixed and permeabilized using Foxp3/Transcription Factor Staining Buffer Set (ThermoFisher Scientific) according to the manufacturer’s instructions. Cells were then stained using a cocktail of metal-conjugated antibodies targeting intracellular antigens (Supplementary Table 3) and incubated for 1 h at ambient temperature. Cells were washed twice with CSB and fixed overnight using 2% formaldehyde in PBS.

For acquisition by CyTOF XT^73^, cells were stained with DNA intercalator (0.125 mM Iridium-191/-193 or MaxPar Intercalator-Ir (Standard BioTools Inc.) in 2% formaldehyde and incubated for 20 min at ambient temperature. Cells were washed twice with CSB followed by two washes with Maxpar Cell Acquisition Solution (CAS) Plus (Standard BioTools Inc.) before being filtered through a 35 mm nylon mesh, diluted to 500,000 cells ml^−1^ using CAS Plus and divided into polypropylene tubes. A total of 2 × 10^6^ cells per tube in pelleted form were then placed in the chilled carousel of the CyTOF XT instrument (Standard BioTools Inc.). EQ Six (EQ6) element calibration beads (Standard BioTools Inc.) were added to a tube and placed in the carousel. The autosampler of the CyTOF XT dispensed CAS Plus to the pelleted sample tubes, mixed with EQ beads 0.1×, and then acquired on CyTOF XT mass cytometers at a rate of 300–500 cells s^−1^ using CyTOF software v.8.0 with noise reduction, event length limits of 10–150 pushes, and a flow rate of 0.030 ml min^−1^.

### Mass cytometry antibodies and reagents

Purified antibodies were obtained in carrier/protein-free buffer and coupled to lanthanide metals using the MaxPar X8 or MCP9 antibody conjugation kits (Standard BioTools Inc.) as per the manufacturer’s recommendations. Metal-conjugated antibodies were also purchased from Standard BioTools. The antibodies used for this study are listed in Supplementary Tables 1–3.

### Mass cytometry data analyses

Samples from participants undergoing sex reassignment therapy were processed through mass cytometry in four batches to investigate immune composition and phenotype. This involved analyzing.fcs files from 60 samples from 20 series of participants receiving testosterone treatment. Data analysis was conducted in R. The data were arcsin h transformed with a cofactor of five using the flowCore package. Beads and dead cells were filtered out. Batches were combined, and batch effects in marker expression were eliminated using the sva package. The resulting matrix was used for immune composition analysis with the FlowSOM package^74^.

Initially, 30 clusters were identified, neutrophil clusters were annotated, and the remaining non-neutrophil cells were clustered into a total of 100 clusters. A total of 113 unique clusters were annotated on the basis of median marker expression using the pheatmap package. A total of 12,377,068 cells from the 60 samples of participants undergoing testosterone treatment were further analysed. This analysis included investigating immune phenotypes using PAGA^75^ (see below) and examining the effects of testosterone on immune cell composition using a mixed-effects model with the lme4 package. For linear mixed-effects models, the frequency of 35 immune subsets was modelled considering visit (baseline, 3 months and 12 months) and age as fixed effects, and participant ID as random effect. Significant visit effects were determined using a *P* value of 0.05 and a 5% FDR threshold, with beta coefficients indicating the directionality of the effect.

### Spectral flow cytometry analysis of AR and ESR expression

For ESRa staining, PBMCs were extracted from heparinized whole blood, as described above. One million live cells were aliquoted per test, washed twice in ice-cold PBS, and incubated with LIVE/DEAD Fixable Blue dye (ThermoFisher Scientific) for 10 min at 4 °C. PBMCs were then washed in ice-cold FACS buffer (2% FBS, 0.5 mM EDTA in PBS) and FcR blocked using an in-house-prepared solution for 10 min at ambient temperature. The Horizon Brilliant Stain Buffer Plus (BD Biosciences) and extracellular antibodies (Supplementary Table 4) were added, and cells were incubated for 30 min at 4 °C followed by fixation and permeabilization with Fixative buffer (Cytodelics AB) or Foxp3/Transcription Factor Staining Buffer Set (ThermoFisher Scientific) according to the manufacturer’s instructions. Cells were mixed with FcR block buffer and, after 10 min at room temperature, intracellular antibodies (Supplementary Table 4) were added, and the samples were incubated for 30 min at 4 °C. For AR staining, WBCs from heparinized whole blood were prepared using a Cytodelics kit, and 1.5 million fixed-permeabilized cells was aliquoted per test and exposed to FcR block (BD Biosciences) for 30 min at 4 °C. Horizon Brilliant Stain Buffer Plus (BD Biosciences) and all-antibody cocktail (Supplementary Table 4) were added, and cells were incubated overnight at 4 °C. AR and ESRa antibody concentrations were established on the cell line MCF7 (ATCC); the specificity of AR antibody was also verified using a competitive displacement approach on MCF7 cells. Briefly, 60,000 cells were collected at passage two, fixed-permeabilized using Cytodelics kit, FcR blocked and stained as described for WBCs. Unconjugated antibodies and isotype controls information is present in Supplementary Table 4. After a wash in cold FACS buffer, data were acquired using an Aurora spectral cytometer (Cytek Biosciences). Cytobank Community (Beckman Coulter) software was used for data analysis.

### PBMC stimulation and intracellular staining by spectral flow cytometry

Cryopreserved PBMCs obtained from individuals undergoing gender-affirming testosterone treatment were collected at baseline and after 3 months of testosterone treatment. These cells were thawed in thawing medium (RPMI 1640 HyClone supplemented with 10% FBS, 1% penicillin-streptomycin and Benzonase-nuclease (Sigma-Aldrich).

The cells were then counted using a Cellaca MX (Nexcelom), plated and incubated for 1 h at 37 °C and 5% CO_2_ to rest. After this, some samples were left untreated while others were stimulated ex vivo with PMA (50 ng ml^−1^) and Ionomycin (1 μg ml^−1^) for 4 h. Brefeldin A (5 μg ml^−1^) and Monensin (2 μg ml^−1^) were added during the last 3 h of stimulation.

Following stimulation, the cells were washed twice in ice-cold PBS and then incubated with LIVE/DEAD Fixable Blue dye for 10 min at 4 °C. The cells were then washed in ice-cold FACS buffer and FcR blocked using blocking solution prepared in-house for 10 min at ambient temperature.

Horizon Brilliant Stain Buffer Plus was added, and the cells were stained with a cocktail of fluorochrome conjugated antibodies targeting surface antigens for 30 min at 4 °C (Supplementary Table 4). The cells were then fixed using Fix, Lysis and Wash buffers (Whole blood processing kit; Cytodelics AB) and permeabilized using permeabilization buffer (ThermoFisher Scientific).

Next, the cells were stained with a cocktail of antibodies targeting intracellular antigens (Supplementary Table 4) for 30 min at 4 °C and then acquired using an Aurora spectral cytometer.

### Plasma protein profiling by Olink

Plasma protein data was generated using the Olink assay, a proximity extension assay (Olink AB)^76^. Plasma (20 μl) from each sample was thawed and analysed using a Target Inflammation panel (Olink AB), at the Affinity Proteomics Stockholm, Science for Life Laboratory or Olink AB. In these assays, plasma proteins are dually recognized by pairs of antibodies coupled to a cDNA-strand that ligates when brought into proximity by its target, extended by a polymerase and detected using a Biomark HD 96.96 dynamic PCR array (Standard BioTools Inc.). Analyses of differentially abundant plasma proteins were performed using linear mixed-effects models considering age as fixed effects.

### Whole blood pretreatment in vitro using testosterone and AR antagonist for Olink analysis

A blood sample obtained from a healthy female donor was mixed in equal ratio with WB-STIM buffer (Cytodelics AB) without phenol red. The sample was then split into three groups: untreated, treated with testosterone (Sigma-Aldrich) alone at 10 ng ml^−1^, or treated with a combination of testosterone and the AR antagonist Enzalutamide (Sigma-Aldrich) at 2.3 μg ml^−1^. All samples were incubated for 28 h at 37 °C and 5% CO_2_. After incubation, supernatants were collected, cryopreserved and later analysed using the Olink Target Inflammation panel (Olink AB) as described above.

### Whole blood pretreatment and stimulation in vitro for Nanostring and mass cytometry analysis

For the in vitro pretreatment step, blood samples were mixed in equal ratio with WB-STIM buffer (Cytodelics AB) without phenol red and split as follows: untreated, treated with DHT (Sigma-Aldrich) alone at 10 ng ml^−1^, treated with DHT combined with Enzalutamide (Sigma-Aldrich) at 2.3 μg ml^−1^ or treated with fulvestrant (Sigma-Aldrich) alone at 100 nM. Samples were incubated for 20 h at 37 °C and 5% CO_2_. DHT was chosen because this androgen cannot be converted to oestradiol by aromatase^77^. Fulvestrant is a degrader of the ESR and blocks oestradiol-mediated signalling^78^.

For Nanostring analyses, blood samples from healthy cisgender female donors (*n* = 11) were pretreated and then immediately stimulated with either LPS (10 ng ml^−1^) or R848 (1 μM) for 3 h or left unstimulated as a control. Samples were then centrifuged at 4 °C and 1,200*g* for 10 min and supernatants were collected, cryopreserved and analysed using SIMOA. The remaining 1 ml of blood was mixed with PAXgene solution (BD Biosciences), incubated for 2 h at ambient temperature and stored at −80 °C. RNA samples were prepared using a QIAcube with the PAXgene blood RNA kit (Qiagen) and analysed using the Nanostring nCounter Sprint Profiler system with a broad human immune response panel (Human Immunology v.2 Gene Expression CodeSet) as described previously^6^. For each sample, 100 ng of total RNA in a final volume of 5 μl was mixed with a capture probe and a reporter probe tagged with a fluorescent barcode from the gene expression code set. Probes and target transcripts were hybridized overnight at 65 °C for around 19 h according to the manufacturer’s recommendations. Hybridized samples were run on the Nanostring nCounter instrument using the corresponding protocol, in which excess capture and reporter probes were removed and transcript-specific ternary complexes were immobilized on the surface of the cartridge. The images from samples were scanned at high resolution by the nCounter instrument and gene expression data were collected after scanning and image processing.

For mass cytometry analyses of cytokine production, blood samples from healthy cisgender females (*n* = 5) of reproductive age were collected before the ovulation phase of the menstrual cycle (day 1–10 from the first day of menstruation), pretreated and then immediately stimulated with either LPS (0.1 ng ml^−1^) or PMA (50 ng ml^−1^) combined with ionomycin (1 μg ml^−1^) for 4 h or left unstimulated as a control. Brefeldin A (5 μg ml^−1^) and Monensin (2 μg ml^−1^) were added in all conditions. Samples were then fixed and lysed using Lysis and Wash buffers (Whole blood processing kit; Cytodelics AB). After fixation/lysis, cells were cryopreserved using CRYO#20 (Cytodelics AB) and analysed using intracellular staining mass cytometry as described above.

### Analyses of Nanostring gene expression data

Batch-normalized data were log-transformed and scaled to have unit variance and zero mean. This was followed by principal component analysis (PCA). The resulting PCAs were then plotted alongside the PCA loadings of hallmark TNF genes.

### Quantification of IFNa and IFNb by Simoa

IFNa subtypes were quantified in plasma and in supernatants of ex vivo*-*stimulated PBMCs using Simoa digital ELISA (Quanterix) with HomeBrew assays as previously described^79^. Several IFNα subtypes were measured using a pan-IFNα subtype assay (Quanterix), with IFNa17 (PBL Assay Science) as a reference standard. Antibodies cloned from two patients with mutated APS1 were used for multi-IFNα subtype quantification. The 8H1 clone was coated on paramagnetic beads and used as the capture antibody (0.1 μg ml^−1^), and the 12H5 clone was biotinylated at a ratio of 30:1 and used as the detector. The limit of detection for IFNα was 0.03 fg ml^−1^. IFNβ was also quantified in plasma from the cohort. For the IFNβ assay, the 710906-9 IFNβ antibody (PBL Assay Science) was coated on paramagnetic beads (0.3 μg ml^−1^) and used as a capture antibody. The 710323-9 antibody (PBL Assay Science) was biotinylated and used as the detector (30:1). Recombinant IFNβ (PBL Assay Science) served as a standard to determine unknown sample concentrations. The LOD for IFNβ was 0.3 pg ml^−1^.

### Reporting summary

Further information on research design is available in the Nature Portfolio Reporting Summary linked to this article.

## Online content

Any methods, additional references, Nature Portfolio reporting summaries, source data, extended data, supplementary information, acknowledgements, peer review information; details of author contributions and competing interests; and statements of data and code availability are available at 10.1038/s41586-024-07789-z.

## Supplementary information

Supplementary Tables Table 1, Panel of antibodies for mass cytometry. Table 2, Antibodies for mass cytometry (extracellular antigens). Table 3, Antibodies for mass cytometry (intracellular antigens). Table 4, Antibodies for flow cytometry.

Reporting Summary

## Data Availability

Raw mass and flow cytometry data (FCS files) are available at FlowReposity.org (https://flowrepository.org/id/FR-FCM-Z75Z). Plasma protein (Olink), induced cytokines (SIMOA), blood mRNA-seq count tables, sc-mRNA-seq count tables as well as ATAC-seq data are available at Zenodo (https://zenodo.org/doi/10.5281/zenodo.11517624)^80^.

## Code availability

All scripts and data for reproduction of figures are available at GitHub (https://github.com/Brodinlab/Gender-affirming-Testosterone-treatment) and at Zenodo (https://zenodo.org/doi/10.5281/zenodo.11517624)^80^.
