# Author Correction: Immune system adaptation during gender-affirming testosterone treatment

**DOI:** 10.1038/s41586-024-08081-w

**Published:** 2024-09-24

**Authors:** Tadepally Lakshmikanth, Camila Consiglio, Fabian Sardh, Rikard Forlin, Jun Wang, Ziyang Tan, Hugo Barcenilla, Lucie Rodriguez, Jamie Sugrue, Peri Noori, Margarita Ivanchenko, Laura Piñero Páez, Laura Gonzalez, Constantin Habimana Mugabo, Anette Johnsson, Henrik Ryberg, Åsa Hallgren, Christian Pou, Yang Chen, Jaromír Mikeš, Anna James, Per Dahlqvist, Jeanette Wahlberg, Anders Hagelin, Mats Holmberg, Marie Degerblad, Magnus Isaksson, Darragh Duffy, Olle Kämpe, Nils Landegren, Petter Brodin

**Affiliations:** 1 Department of Women’s and Children’s Health, Karolinska Institutet, Solna, Sweden; 2 Department of Laboratory Medicine, Lund University, Lund, Sweden; 3 Center for Molecular Medicine, Department of Medicine, Karolinska Institutet, Solna, Sweden; 4 Science for Life Laboratory, Department of Medical Biochemistry and Microbiology, Uppsala University, Uppsala, Sweden; 5 Translational Immunology Unit, Institut Pasteur, Paris, France; 6 Department of Clinical Chemistry, Sahlgrenska University Hospital, Gothenburg, Sweden; 7 Department of Internal Medicine and Clinical Nutrition, University of Gothenburg, Gothenburg, Sweden; 8 Department of Public Health and Clinical Medicine, Umeå University, Umeå, Sweden; 9 Department of Medicine, Örebro University, Örebro, Sweden; 10 ANOVA, Karolinska University Hospital, Stockholm, Sweden; 11 Department of Medicine, Karolinska Institutet, Stockholm, Sweden; 12 Department of Molecular Medicine and Surgery, Karolinska Institutet, Solna, Sweden; 13 Department of Medical Sciences, Uppsala University, Uppsala, Sweden; 14 Department of Endocrinology, Metabolism and Diabetes, Karolinska University Hospital, Stockholm, Sweden; 15 Medical Research Council, Laboratory of Medical Sciences, London, UK; 16 Department of Immunology and Inflammation, Imperial College London, London, UK

**Keywords:** Translational research, Epigenetics in immune cells

Correction to: *Nature* 10.1038/s41586-024-07789-z Published online 4 September 2024

In the version of the article initially published, the *y*-axis labels in Fig. 2b originally read “WBCs (%)” but have now been amended to “pDC of WBC (%)”, “CD8^+^ MAIT of WBC (%)” and “CD24^+^CD8^+^ T_CM_ of WBC (%)” in the HTML and PDF versions of the article.

