# Supplementary material for: Immune system adaptation during gender-affirming testosterone treatment
Source: Nature. 2024 Sep 4;633(8028):155–64. doi: 10.1038/s41586-024-07789-z (PMC11374716; doi:10.1038/s41586-024-07789-z)
Supplement: Supplementary file 1 — Table 1, Panel of antibodies for mass cytometry. Table 2, Antibodies for mass cytometry (extracellular antigens). Table 3, Antibodies for mass cytometry (intracellular antigens). Table 4, Antibodies for flow cytometry. [file 41586_2024_7789_MOESM1_ESM.pdf]

nature portfolio

https://doi.org/10.1038/s41586-024-07789-z

Supplementary information

Immune system adaptation during gender-affirming testosterone treatment

In the format provided by the authors and unedited

Table 1. Broad extension panel of antibodies used in mass cytometry.

Metal tag	Marker	Catalog number	Antibody dilution, times	Clone	Vendor*
89Y	CD45	3089003B	200	HI30	Standard BioTools
102Pd	Barcode	201060	-	-	Standard BioTools
104Pd	Barcode	201060	-	-	Standard BioTools
105Pd	Barcode	201060	-	-	Standard BioTools
106Pd	Barcode	201060	-	-	Standard BioTools
108Pd	Barcode	201060	-	-	Standard BioTools
110Cd	CD33	303402	125	WM53	BioLegend
111Cd	CD26	302702	60	BA5b	BioLegend
112Cd	CD11c	337202	60	Bu15	BioLegend
113Cd	IgD	348202	250	IA6-2	BioLegend
114Cd	HLA-DR	307602	125	L243	BioLegend
115In	CD57	322302	200	HCD57	BioLegend
140Ce	CD71	334102	200	CY1G4	BioLegend
141Pr	CD49d	3141004B	100	9F10	Standard BioTools
142Nd	CD43	14-0439-82	125	84-3C1	eBiosciences
143Nd	CD3e	317302	250	UCHT1	BioLegend
144Nd	CD45RB	310202	125	MEM-55	BioLegend
145Nd	CD81	349502	60	5A6	BioLegend
146Nd	CD52	316002	125	HI186	BioLegend
147Sm	CD1c	331502	125	L161	BioLegend
148Nd	CD55	311302	125	JS11	BioLegend
149Sm	CD25	3149010B	100	2A3	Standard BioTools
150Nd	CD64	305002	60	10.1	BioLegend
151Eu	CD123	306002	100	6H6	BioLegend
152Sm	TCRgd	TCR1061	125	5A6.E9	Thermo Fisher Scientific
153Eu	Siglec-8	837535	125	837535	R&D Systems
154Sm	CD95	305602	125	DX2	BioLegend
155Gd	CD73	344002	60	AD2	BioLegend
156Gd	CD20	302302	200	2H7	BioLegend
157Gd	CD9	14-0098-82	75	SN4 C3-3A2	eBiosciences
158Gd	CD34	343502	30	581	BioLegend
159Tb	CD22	302502	60	HIB22	BioLegend
160Gd	CD14	301802	100	M5E2	BioLegend
161Dy	CD161	339902	100	HP-3G10	BioLegend
162Dy	CD29	303002	100	TS2/16	BioLegend
163Dy	4-1BB	309802	125	4B4-1	BioLegend
164Dy	CD62L	304802	125	DREG-56	BioLegend
165Ho	CD127	3165008B	100	A019D5	Standard BioTools
166Er	CD24	311102	40	ML5	BioLegend
167Er	CD27	3167006B	100	L128	BioLegend

168Er	CD141	344102	60	M80	BioLegend
169Tm	CD45RA	3169008B	200	HI100	Standard BioTools
170Er	CD38	303502	60	HIT2	BioLegend
171Yb	CD85j	333702	60	GHI/75	BioLegend
172Yb	CD103	350202	60	Ber-ACT8	BioLegend
173Yb	CD56	559043	150	NCAM16.2	BD Biosciences
174Yb	CD99	318002	60	HCD99	BioLegend
175Lu	CD28	302902	60	CD28.2	BioLegend
176Yb	CD39	328202	60	A1	BioLegend
191Ir	DNA Ir	201192A	1000	Cell-ID DNA Intercalator	Standard BioTools
193Ir	DNA Ir	201192A	1000	Cell-ID DNA Intercalator	Standard BioTools
194Pt	CD8a	344702	50	SK1	BD Biosciences
195Pt	CD5	300602	50	UCHT2	BioLegend
196Pt	CD7	343102	200	CD7-6B7	BioLegend
198Pt	CD4	300502	85	RPA-T4	BioLegend
209Bi	CD16	3209002B	100	3G8	Standard BioTools

**All antibodies that are not from Standard BioTools were purchased in a purified format and coupled In-house.*

Table 2. Surface staining panel of antibodies used in intracellular mass cytometry.

Metal tag	Marker	Catalog number	Antibody dilution, times	Clone	Vendor*
89Y	CD45	3089003B	200	HI30	Standard BioTools
102Pd	Barcode	201060	-	-	Standard BioTools
104Pd	Barcode	201060	-	-	Standard BioTools
105Pd	Barcode	201060	-	-	Standard BioTools
106Pd	Barcode	201060	-	-	Standard BioTools
108Pd	Barcode	201060	-	-	Standard BioTools
112Cd	CD11c	337202	60	Bu15	BioLegend
114Cd	HLA-DR	307602	125	L243	BioLegend
142Nd	CD19	3142001B	100	HIB19	BioLegend
143Nd	CD3e	317302	250	UCHT1	BioLegend
145Nd	CD81	349502	60	5A6	BioLegend
147Sm	CD1c	331502	125	L161	BioLegend
151Eu	CD123	306002	100	6H6	BioLegend
153Eu	Siglec-8	837535	125	837535	R&D Systems
157Gd	CD9	14-0098-82	75	SN4 C3-3A2	eBiosciences
160Gd	CD14	301802	100	M5E2	BioLegend
161Dy	CD161	339902	100	HP-3G10	BioLegend
162Dy	SLAMF7	331802	100	162.1	BioLegend
167Er	CD27	3167006B	100	L128	BioLegend
168Er	CD141	344102	60	M80	BioLegend
169Tm	CD45RA	3169008B	200	HI100	Standard BioTools
173Yb	CD56	559043	150	NCAM16.2	BD Biosciences
191Ir	DNA Ir	201192A	1000	Cell-ID DNA Intercalator	Standard BioTools
193Ir	DNA Ir	201192A	1000	Cell-ID DNA Intercalator	Standard BioTools
194Pt	CD8a	344702	50	SK1	BD Biosciences
195Pt	CD5	300602	50	UCHT2	BioLegend
196Pt	CD7	343102	200	CD7-6B7	BioLegend
198Pt	CD4	300502	85	RPA-T4	BioLegend
209Bi	CD16	3209002B	100	3G8	Standard BioTools

**All antibodies that are not from Standard BioTools were purchased in a purified format and coupled in-house.*

Table 3. Intracellular staining panel of antibodies used in mass cytometry.

Metal tag	Marker	Catalog number	Antibody dilution, times	Clone	Vendor*
149Sm	IL-4	500802	75	MP4-25D2	BioLegend
150Nd	IFNγ	506502	125	B27	BioLegend
156Gd	IL-6	3156011B	100	MQ2-13AS	Standard BioTools
159Tb	IL-1ß	508201	60	JK1B-1	BioLegend
175Lu	TNFα	502941	75	Mab11	BioLegend

**All antibodies that are not from Standard BioTools were purchased in a purified format and coupled in-house.*

Table 4. Fluorescent marker antibodies (Surface and intracellular) used in spectral flow cytometry.

Fluorophore	Marker	Catalog number	Antibody dilution, times	Clone	Vendor*
BUV496	HLA-DR	753685	100	L243	BD Biosciences
BUV737	CD56	612767	100	NCAM16.2	BD Biosciences
BUV805	CD8	612890	100	SK1	BD Biosciences
BV421	CD123	306018	100	6H6	BioLegend
eF450	CD15	48-0158-41	33	MMA	Invitrogen
BV570	CD16	302035	100	3G8	BioLegend
FITC	CD4	300505	62,5	RPA-T4	BioLegend
Spark Blue 574	CD3	300487	100	UCHT1	BioLegend
BB630-P2	CD19	624294	100	SJ25C1	BD Biosciences
BB790-P	CD14	624296	100	M5E2	BD Biosciences
RB780	CD14	569069	100	M5E2	BD Biosciences
PE	AR*	IC5876P	200	523339	R&D Systems
PE	-	IC0041P	40	Mouse IgG2B – Isotype control	R&D Systems
APC	AR*	IC5876A	50	523339	R&D Systems
PE	ESRα#	ab209288	5000	E115	Abcam
PE	-	ab37407	1250	Rabbit IgG – Isotype control	Abcam
BV421	IFNγ	506538	350	B27	BioLegend
PE-Cy7	CCR7	353226	100	G043H7	BioLegend
cFluor R685	CD45RA	RC-00656	100	HI100	Cytek Biosciences
APC	CD45RA	304112	100	HI100	BioLegend

**AR, androgen receptor; #ESRα, estrogen receptor alpha.*
