# Supplementary material for: Immune system adaptation during gender-affirming testosterone treatment
Source: Nature. 2024 Sep 4;633(8028):155–64. doi: 10.1038/s41586-024-07789-z (PMC11374716; doi:10.1038/s41586-024-07789-z)
Supplement: Supplementary file 2 — Reporting Summary [file 41586_2024_7789_MOESM2_ESM.pdf]

nature portfolio

Corresponding author(s): Petter Brodin

Last updated by author(s): Jun 6, 2024

Reporting Summary

Nature Portfolio wishes to improve the reproducibility of the work that we publish. This form provides structure for consistency and transparency in reporting. For further information on Nature Portfolio policies, see our Editorial Policies and the Editorial Policy Checklist.

Statistics

For all statistical analyses, confirm that the following items are present in the figure legend, table legend, main text, or Methods section.

n/a	Confirmed	
☐	☒	The exact sample size (n) for each experimental group/condition, given as a discrete number and unit of measurement
☐	☒	A statement on whether measurements were taken from distinct samples or whether the same sample was measured repeatedly
☐	☒	The statistical test(s) used AND whether they are one- or two-sided<br>*Only common tests should be described solely by name; describe more complex techniques in the Methods section.*
☐	☒	A description of all covariates tested
☐	☒	A description of any assumptions or corrections, such as tests of normality and adjustment for multiple comparisons
☐	☒	A full description of the statistical parameters including central tendency (e.g. means) or other basic estimates (e.g. regression coefficient) AND variation (e.g. standard deviation) or associated estimates of uncertainty (e.g. confidence intervals)
☐	☒	For null hypothesis testing, the test statistic (e.g. F, t, r) with confidence intervals, effect sizes, degrees of freedom and P value noted<br>*Give P values as exact values whenever suitable.*
☒	☐	For Bayesian analysis, information on the choice of priors and Markov chain Monte Carlo settings
☒	☐	For hierarchical and complex designs, identification of the appropriate level for tests and full reporting of outcomes
☒	☐	Estimates of effect sizes (e.g. Cohen's d, Pearson's r), indicating how they were calculated

Our web collection on statistics for biologists contains articles on many of the points above.

Software and code

Policy information about availability of computer code

Data collection	No software was used for data collection.
Data analysis	All custom code in GitHub: https://github.com/Brodinlab/Gender-affirming-Testosterone-treatment. CyTOF software v8.1 (Standard Biotools) , Cytobank Community v10.3 (Beckman Dickinson) and FlowJo 10.10.0 (BD Biosciences).

For manuscripts utilizing custom algorithms or software that are central to the research but not yet described in published literature, software must be made available to editors and reviewers. We strongly encourage code deposition in a community repository (e.g. GitHub). See the Nature Portfolio guidelines for submitting code & software for further information.

Data

Policy information about availability of data

All manuscripts must include a data availability statement. This statement should provide the following information, where applicable:

- Accession codes, unique identifiers, or web links for publicly available datasets
- A description of any restrictions on data availability
- For clinical datasets or third party data, please ensure that the statement adheres to our policy

Raw mass and flow cytometry data (FCS-files) are available at FlowReposity.org (https://flowrepository.org/id/FR-FCM-Z75Z). Plasma protein (Olink), induced cytokines (SIMOA), blood mRNA-sequencing count tables, single-cell mRNA-sequencing count tables as well as ATAC-seq. data is available via Zenodo: https://zenodo.org/doi/10.5281/zenodo.1151762421.

Research involving human participants, their data, or biological material

Policy information about studies with human participants or human data. See also policy information about sex, gender (identity/presentation), and sexual orientation and race, ethnicity and racism.

Reporting on sex and gender	Sex and gender are important considerations for the study and study participants have been described with respect to sex and gender in accordance with Nature policies and with careful consideration of language acceptable to the trans community. The co-authors have consulted representatives of the trans community in planning the study and preparing the manuscript.
Reporting on race, ethnicity, or other socially relevant groupings	Race and ethnicity are not reported.
Population characteristics	Age and sex is reported and taken into account into mixed effects models and described when appropriate.
Recruitment	23 adult individuals who were assigned female at birth and were undergoing masculinizing gender-affirming treatment were enrolled at specialist centers for transgender medicine in Stockholm, Uppsala, Linköping, and Umeå in Sweden between 2016 and 2023.
Ethics oversight	Swedish Ethical Review Authority (2016/1422-31/1).

Note that full information on the approval of the study protocol must also be provided in the manuscript.

Field-specific reporting

Please select the one below that is the best fit for your research. If you are not sure, read the appropriate sections before making your selection.

[x] Life sciences [ ] Behavioural & social sciences [ ] Ecological, evolutionary & environmental sciences

For a reference copy of the document with all sections, see nature.com/documents/nr-reporting-summary-flat.pdf

Life sciences study design

All studies must disclose on these points even when the disclosure is negative.

Sample size	The number of included subjects was maximized but not pre-specified by a priori power analysis.
Data exclusions	No exclusions
Replication	All in vitro experiments performed on healthy donor blood samples were performed repeatedly to ensure reproducibility and all replicates reported.
Randomization	This was an observational study and not a treatment investigation, thus randomization was not performed.
Blinding	Blinded therapy would not have been possible given the striking and well-established consequences of testosterone therapy.

Reporting for specific materials, systems and methods

We require information from authors about some types of materials, experimental systems and methods used in many studies. Here, indicate whether each material, system or method listed is relevant to your study. If you are not sure if a list item applies to your research, read the appropriate section before selecting a response.

Materials & experimental systems

n/a	Involved in the study	
[ ]	[x]	Antibodies
[x]	[ ]	Eukaryotic cell lines
[x]	[ ]	Palaeontology and archaeology
[x]	[ ]	Animals and other organisms
[x]	[ ]	Clinical data
[x]	[ ]	Dual use research of concern
[x]	[ ]	Plants

Methods

n/a	Involved in the study	
[x]	[ ]	ChIP-seq
[ ]	[x]	Flow cytometry
[x]	[ ]	MRI-based neuroimaging

Antibodies

Antibodies used	Table 1. Broad extension panel of antibodies used in mass cytometry.

Metal tag Marker Catalog number Antibody dilution, times Clone Vendor*
89Y CD45 3089003B 200 HI30 Standard BioTools
102Pd Barcode 201060 - - Standard BioTools
104Pd Barcode 201060 - - Standard BioTools
105Pd Barcode 201060 - - Standard BioTools
106Pd Barcode 201060 - - Standard BioTools
108Pd Barcode 201060 - - Standard BioTools
110Cd CD33 303402 125 WM53 BioLegend
111Cd CD26 302702 60 BA5b BioLegend
112Cd CD11c 337202 60 Bu15 BioLegend
113Cd IgD 348202 250 IA6-2 BioLegend
114Cd HLA-DR 307602 125 L243 BioLegend
115In CD57 322302 200 HCD57 BioLegend
140Ce CD71 334102 200 CY1G4 BioLegend
141Pr CD49d 3141004B 100 9F10 Standard BioTools
142Nd CD43 14-0439-82 125 84-3C1 eBiosciences
143Nd CD3e 317302 250 UCHT1 BioLegend
144Nd CD45RB 310202 125 MEM-55 BioLegend
145Nd CD81 349502 60 5A6 BioLegend
146Nd CD52 316002 125 HI186 BioLegend
147Sm CD1c 331502 125 L161 BioLegend
148Nd CD55 311302 125 JS11 BioLegend
149Sm CD25 3149010B 100 2A3 Standard BioTools
150Nd CD64 305002 60 10.1 BioLegend
151Eu CD123 306002 100 6H6 BioLegend
152Sm TCRgd TCR1061 125 5A6.E9 Thermo Fisher Scientific
153Eu Siglec-8 837535 125 837535 R&D Systems
154Sm CD95 305602 125 DX2 BioLegend
155Gd CD73 344002 60 AD2 BioLegend
156Gd CD20 302302 200 2H7 BioLegend
157Gd CD9 14-0098-82 75 SN4 C3-3A2 eBiosciences
158Gd CD34 343502 30 581 BioLegend
159Tb CD22 302502 60 HIB22 BioLegend
160Gd CD14 301802 100 M5E2 BioLegend
161Dy CD161 339902 100 HP-3G10 BioLegend
162Dy CD29 303002 100 TS2/16 BioLegend
163Dy 4-1BB 309802 125 4B4-1 BioLegend
164Dy CD62L 304802 125 DREG-56 BioLegend
165Ho CD127 3165008B 100 A019D5 Standard BioTools
166Er CD24 311102 40 ML5 BioLegend
167Er CD27 3167006B 100 L128 BioLegend
168Er CD141 344102 60 M80 BioLegend
169Tm CD45RA 3169008B 200 HI100 Standard BioTools
170Er CD38 303502 60 HIT2 BioLegend
171Yb CD85j 333702 60 GHI/75 BioLegend
172Yb CD103 350202 60 Ber-ACT8 BioLegend
173Yb CD56 559043 150 NCAM16.2 BD Biosciences
174Yb CD99 318002 60 HCD99 BioLegend
175Lu CD28 302902 60 CD28.2 BioLegend
176Yb CD39 328202 60 A1 BioLegend
191Ir DNA Ir 201192A 1000 Cell-ID DNA Intercalator Standard BioTools
193Ir DNA Ir 201192A 1000 Cell-ID DNA Intercalator Standard BioTools
194Pt CD8a 344702 50 SK1 BD Biosciences
195Pt CD5 300602 50 UCHT2 BioLegend
196Pt CD7 343102 200 CD7-6B7 BioLegend
198Pt CD4 300502 85 RPA-T4 BioLegend
209Bi CD16 3209002B 100 3G8 Standard BioTools
*All antibodies that are not from Standard BioTools were purchased in a purified format and coupled
In-house.

Table 2. Surface staining panel of antibodies used in intracellular mass cytometry.
Metal tag Marker Catalog number Antibody dilution, times Clone Vendor*
89Y CD45 3089003B 200 HI30 Standard BioTools
102Pd Barcode 201060 - - Standard BioTools
104Pd Barcode 201060 - - Standard BioTools
105Pd Barcode 201060 - - Standard BioTools
106Pd Barcode 201060 - - Standard BioTools
108Pd Barcode 201060 - - Standard BioTools

112Cd CD11c 337202 60 Bu15 BioLegend
114Cd HLA-DR 307602 125 L243 BioLegend
142Nd CD19 3142001B 100 HIB19 BioLegend
143Nd CD3e 317302 250 UCHT1 BioLegend
145Nd CD81 349502 60 5A6 BioLegend
147Sm CD1c 331502 125 L161 BioLegend
151Eu CD123 306002 100 6H6 BioLegend
153Eu Siglec-8 837535 125 837535 R&D Systems
157Gd CD9 14-0098-82 75 SN4 C3-3A2 eBiosciences
160Gd CD14 301802 100 M5E2 BioLegend
161Dy CD161 339902 100 HP-3G10 BioLegend
162Dy SLAMF7 331802 100 162.1 BioLegend
167Er CD27 3167006B 100 L128 BioLegend
168Er CD141 344102 60 M80 BioLegend
169Tm CD45RA 3169008B 200 HI100 Standard BioTools
173Yb CD56 559043 150 NCAM16.2 BD Biosciences
191Ir DNA Ir 201192A 1000 Cell-ID DNA Intercalator Standard BioTools
193Ir DNA Ir 201192A 1000 Cell-ID DNA Intercalator Standard BioTools
194Pt CD8a 344702 50 SK1 BD Biosciences
195Pt CD5 300602 50 UCHT2 BioLegend
196Pt CD7 343102 200 CD7-6B7 BioLegend
198Pt CD4 300502 85 RPA-T4 BioLegend
209Bi CD16 3209002B 100 3G8 Standard BioTools
*All antibodies that are not from Standard BioTools were purchased in a purified format and coupled in-house.
Table 3. Intracellular staining panel of antibodies used in mass cytometry.
Metal tag Marker Catalog number Antibody dilution, times Clone Vendor*
149Sm IL-4 500802 75 MP4-25D2 BioLegend
150Nd IFNγ 506502 125 B27 BioLegend
156Gd IL-6 3156011B 100 MQ2-13AS Standard BioTools
159Tb IL-1ß 508201 60 JK1B-1 BioLegend
175Lu TNF 502941 75 Mab11 BioLegend
*All antibodies that are not from Standard BioTools were purchased in a purified format and coupled in-house.
Table 4. Fluorescent marker antibodies (Surface and intracellular) used in spectral flow cytometry.
Fluorophore Marker Catalog number Antibody dilution, times Clone Vendor*
BUV496 HLA-DR 753685 100 L243 BD Biosciences
BUV737 CD56 612767 100 NCAM16.2 BD Biosciences
BUV805 CD8 612890 100 SK1 BD Biosciences
BV421 CD123 306018 100 6H6 BioLegend
eF450 CD15 48-0158-41 33 MMA Invitrogen
BV570 CD16 302035 100 3G8 BioLegend
FITC CD4 300505 62,5 RPA-T4 BioLegend
Spark Blue 574 CD3 300487 100 UCHT1 BioLegend
BB630-P2 CD19 624294 100 SJ25C1 BD Biosciences
BB790-P CD14 624296 100 M5E2 BD Biosciences
RB780 CD14 569069 100 M5E2 BD Biosciences
PE AR* IC5876P 200 523339 R&D Systems
PE - IC0041P 40 Mouse IgG2B –
Isotype control R&D Systems
APC AR* IC5876A 50 523339 R&D Systems
PE ESR# ab209288 5000 E115 Abcam
PE - ab37407 1250 Rabbit IgG –
Isotype control Abcam
BV421 IFNγ 506538 350 B27 BioLegend
PE-Cy7 CCR7 353226 100 G043H7 BioLegend
cFluor R685 CD45RA RC-00656 100 HI100 Cytek Biosciences
APC CD45RA 304112 100 HI100 BioLegend
*AR, androgen receptor; #ESR, estrogen receptor alpha.

Validation

Specificity testing of 1-3 target cell types with either single- or multi-color analysis (including positive and negative cell types). Once specificity is confirmed, each new lot must perform with similar intensity to the in-date reference lot. Brightness (MFI) is evaluated from both positive and negative populations. Each lot product is validated by QC testing with a series of titration dilutions. Independent validation was performed by fluorescence/mass-minus one, negative and positive controls and where possible such as MCF7 cells (human breast adenocarcinoma cell line) expressing ESR and AR.

Plants

Seed stocks	N/A
Novel plant genotypes	N/A
Authentication	N/A

Flow Cytometry

Plots

Confirm that:

- [x] The axis labels state the marker and fluorochrome used (e.g. CD4-FITC).
- [x] The axis scales are clearly visible. Include numbers along axes only for bottom left plot of group (a 'group' is an analysis of identical markers).
- [x] All plots are contour plots with outliers or pseudocolor plots.
- [x] A numerical value for number of cells or percentage (with statistics) is provided.

Methodology

Sample preparation	For Mass cytometry experiments, whole blood stabilized at blood collection is used and for in vitro stimulation and hormone treatment experiments, whole blood or PBMCs were used. Details are described in supplementary methods.
Instrument	CyTOF XT (Standard BioTools), Aurora spectral flow cytometer (Cytek Biosciences).
Software	CyTOF software v8.1 (Standard Biotools) was used for acquisition of mass cytometry data. Cytobank Community v10.3 (Beckman Dickinson) and FlowJo 10.10.0 (BD Biosciences) were used for manual gating and plotting. For modeling and statistical analyses custom R scripts were used and are available through GitHub: https://github.com/Brodinlab/Gender-affirming-Testosterone-treatment.
Cell population abundance	N/A
Gating strategy	Gating templates are shared via FlowRepository.org and for pre-DC gating it is shared in Extended Data Fig. 2c

- [x] Tick this box to confirm that a figure exemplifying the gating strategy is provided in the Supplementary Information.
